# Meta-analysis of homocysteine-related factors on the risk of colorectal cancer

**DOI:** 10.18632/oncotarget.25355

**Published:** 2018-05-22

**Authors:** S. Pamela K. Shiao, Amanda Lie, Chong Ho Yu

**Affiliations:** ^1^ College of Nursing and Medical College of Georgia, Augusta University, Augusta, GA, USA; ^2^ Citrus Valley Health Partners, Foothill Presbyterian Hospital, Glendora, CA, USA; ^3^ University of Phoenix, Pasadena, CA, USA

**Keywords:** homocysteine, meta-analysis, colorectal cancer, one-carbon metabolism pathway, B vitamins

## Abstract

The major objective of this meta-analysis was to examine the association between homocysteine and related measurements with the risk of colorectal cancer (CRC) and adenomatous polyps (AP). Many studies presented an association between *methyltetrahydrofolate reductase (MTHFR)* gene polymorphisms and risk of CRC. Yet, there have been variances on what homocysteine-related and dietary factors play on the risk of CRC or AP, in association with folate-related one carbon metabolism pathways. We pooled analyses to examine comprehensively all homocysteine related factors including blood tests measurements, dietary, and lifestyle factors for their associations with the risk of CRC and AP. We located 86 articles published from 1995 to 2017. The results revealed that elevated homocysteine levels and decreased vitamin B12 levels in the blood were associated with increased risks of CRC and AP, with case-control studies having greater significant effect sizes compared to that of cohort-control studies. Decreased methionine and vitamin B6 levels in the blood increased the risk of CRC. *MTHFR 677* TT and CT polymorphisms were interacting with elevated homocysteine levels to increase the risk of CRC. Decreased dietary fiber, methionine, vitamin B9 or folate, and vitamin B6 intakes were associated with increased risks of CRC; whereas, increased dietary B12 intake, alcohol intake, and smoking were associated with increased risk of CRC. Further studies can be conducted to examine the mechanistic differences of blood levels of homocysteine-related and dietary factors, including different types of dietary fiber, for their effects on decreasing the homocysteine toxicity to prevent CRC.

## INTRODUCTION

Colorectal cancer (CRC) is the third most common cancer diagnosed in the United States, and the third leading cause of cancer-related deaths in both men and women [1, 2]. Chronic inflammation is a major risk factor for colon and rectum health, that underlies the development of CRC and adenomatous polyps (AP) [3]. Hyperhomocysteinemia (>12–15 μmol/L) is highly prevalent in patients with inflammatory bowels [4, 5], resulted from either decreased absorption or increased requirements for folate (vitamin B9) and other related B vitamins [B2 (riboflavin), B6 (pyridoxine), and B12 (cobalamin)] that are all required for one carbon metabolism (OCM) pathways and homocysteine metabolism [6–10]. Elevated homocysteine level is an independent predictor for all-cause mortality [11–12] and it compromises health of all organ systems [13–16], affecting epigenetic changes for DNA synthesis and healthy living. For each 5 μmol/L homocysteine increment, the risk of mortality increased 32%, and the risk of heart disease increased 52% [11].

When gene mutations in the OCM pathway occur, such as with the *methylenetetra-hydrofolate reductase (MTHFR)* C677T (rs 1801133) polymorphism, there is a deficiency in the methyl-folate enzyme and the activity in the OCM pathway is impaired [8, 9, 16–18]. The *MTHFR* gene is known to be associated with many chronic diseases, including CRC [6–8]. And, *MTHFR* and other genes in the OCM pathway play important roles in DNA methylation, a key mechanism in epigenetics, and more specifically nutrigenomics within the OCM pathway [6–8]. However, an increase in methyl donors such as vitamin B2, B6, B9, B12, or methionine, may help compensate the deficiency of the enzymes in OCM pathways during DNA methylation, synthesis and repair, thus preventing carcinogenesis [19, 20].

Six previous meta-analyses were published on the effects of diet and OCM factors with the risks of cancer. Two of the six meta-analyses included the effects of folate deficiency [9] and hyperhomocysteinemia [21] on the risk of multiple cancers. Three meta-analyses were focused on the effects of dietary folate [7], dietary fiber [22], and dietary supplements [23] on the risk of CRC. These studies concluded that increased homocysteine levels and decreased folate levels in the blood were associated with increased CRC risk; whereas, multivitamins and calcium supplements were beneficial against CRC risk. The sixth and most current meta-analysis pooled analyses on 8 studies for the inflammatory potential of dietary factors on CRC risk [24]. Foods with higher inflammatory dietary index included refined or processed foods and red meat. Anti-inflammatory foods included fruits, vegetables, fish, whole grains, and nuts [24, 25]. Hyperhomocysteinemia and low levels of B vitamins were associated with higher levels of oxidative stress and induction of the inflammatory responses, thus increasing the risk of CRC [3, 26, 27].

In 2007, the American Institute for Cancer Research (AICR) published a comprehensive report providing a major review of the evidence on food, nutrition, physical activity and cancer. They provided convincing evidence that beneficial factors such as physical activity decreased the risk of CRC; whereas, risk factors such as red or processed meat, alcohol, elevated body fat and abdominal fatness, and adult attained height all contributed to increased risk of CRC [28]. Additional studies with convergent findings presented that increased alcohol consumption and foods high in saturated fats increased CRC risk; while higher methionine, vegetables, fiber and folate intake had protective effects against CRC [20, 29–31]. However, summative evidence on various foods and nutrients for CRC prevention [20] presented inconclusive evidence on the effects of vitamin B2 and B12 on CRC risk. To date, there has not been a comprehensive meta-analysis on homocysteine-related measurements including blood tests, dietary, and lifestyle factors in association with the risk of CRC. Therefore, to fill the knowledge gap in understanding about homocysteine and CRC risks, the purpose of this study is to pool all studies with homocysteine-related measurements including blood tests, and dietary and lifestyle factors, for their associations with the risk of CRC.

## RESULTS

### Characteristic of studies

**Figure 1:**
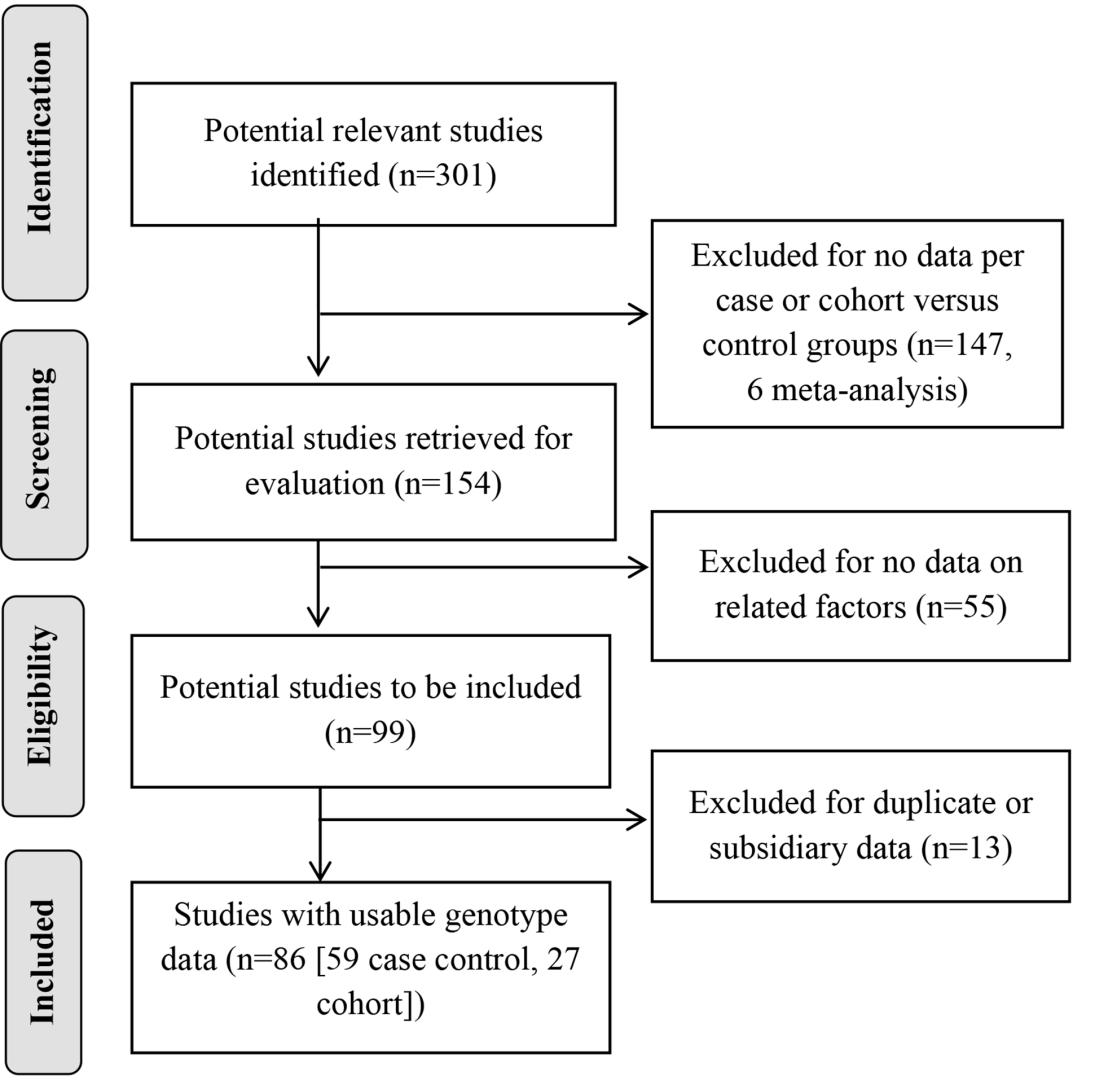
Progression on the selection of studies for the meta-analysis

The progression on the selection of studies is summarized in Figure 1. A total of 86 articles were identified between the years of 1995 to 2017; according to colon health-disease types, 63 papers focused on CRC only, 5 papers reported on both CRC and AP, and 18 papers reported with AP cases only. Each paper was coded by country, ethnicity and cancer sites. Data were grouped per blood tests, dietary or lifestyle factors, and by types of study design (case-control, cohort, or randomized-controlled trial [RCT]) (Supplementary Table 1A). Cancer sites were noted for colon only, rectum only, colorectal combined, colon and rectum sites each, and proximal or distal sites.

When extracting data from each paper, we separated studies for additional subgroups of case types, cancer sites, gender, and racial status when available, which yielded an additional 19 studies for a total of 105 study groups. Specifically, 1 paper included data with one additional study group (population and clinic based) [32]; 5 papers included data by case types of CRC and AP [33–37], 1 of which provided data for 5 groups including CRC, adenomas, polyps, AP combined, and CRC and AP combined [35]; 2 papers included data by cancer sites (colon and rectum separated) [38, 39]; 6 papers included data by gender groups (male and female) [40–45] with 1 of which also included male and female combined [41]; and 1 paper included data for 2 racial groups (Caucasian and African American) [46].

A total of 8,401 cases and 11,009 controls were included in 32 papers (37 study groups) that included homocysteine-related blood measurements, and had data needed for effect size (ES) analysis. These factors included homocysteine, vitamin B12, methionine, vitamin B9 (folate), vitamin B6, and vitamin B2 (ordered by the most number of studies with relevance). A total of 14,900 cases and 149,950 controls were included in 47 papers (60 study groups) that included dietary factors; 31 papers (40 study groups) had data for ES analysis with means and standard deviations (SD), and 34 papers (42 study groups) for relative risk (RR) analysis with the counts per groups. Dietary factors included vitamin B12, methionine, vitamin B2, fiber, vegetables, red meat, multivitamins, and folate (vitamin B9) and vitamin B6, from food sources and/or dietary supplements. Lifestyle factors included alcohol consumption and smoking. A total of 26,107 cases and 163,231 controls were included in 63 papers (73 study groups) that included lifestyle factors; 20 papers (25 study groups) had data for ES analysis and 54 papers (61 study groups) had data for RR analysis. Additionally, 4 studies included data on *MTFHR* 677 gene polymorphism and blood homocysteine levels for CRC; however, only 2 studies [47, 48] were analyzed for ES due to other 2 studies had missing case group and/or SDs (Supplementary Table 1B).

These studies included populations drawn from countries and continents across the globe (Australia, Europe, North America, South America, and Asia and Africa). We checked the racial and ethnic compositions included in each study to be sure that we have properly accounted data from distinct groups versus mixed racial/ethnic groups. The most investigated racial/ethnic populations in these studies were Caucasians (41 studies) and Europeans (35 studies), followed by Asians (23 studies which included 20 East Asians and 3 South Asians), then Middle-Easterners (4 studies), African Americans (1 study), and Hispanics (1 study).

### Pooled analyses

We pooled analyses per categories of blood, dietary, and lifestyle factors in relation to the risk of CRC. Each factor was analyzed by case-control and cohort studies combined and individually, CRC and AP combined and individually, colon and rectum individually, colorectal combined, and for various ethnic groups. Significant ES calculated from mean and SD parameters per groups, and RR generated from frequency counts per groups are presented in the Schema Tables 1–3 per factors, with detailed pooled analyses per factors presented in the Supplementary Tables 2–4B.

**Table 1:** Schema of significant blood tests measurements on the risk of colorectal cancer (CRC) and adenomas/polyps (AP): Effect sizes per case/control and cohort/control study designs and ethnic subgroups

Number of studies (n Case/n Control)	Homocysteine		B12	B9		Methionine	B6
	CRC	AP	CRC	CRC	AP	CRC	--
Case/Control	10 studies (2,458/2981) 0.71^****^	9 studies (1,486/2,061) 1.13^***^	6 studies (774/1,164) –0.99^**^	NS	6 studies (782/1,031) **NS**	2 studies (1,980/3,513) –0.29^*^	--
***Subgroups***							
CR (Colorectal)	7 studies (1,461/1,951) 0.11^**^ 4 European, 3 East Asian	--	--	--	--	--	--
European	7 studies (1,478/1,915) 0.76^****^	4 studies (268/435) 2.27^**^	5 studies (579/969) –1.21^*^	--	NS	2 studies (1,980/3,513) –0.29^*^	--
Caucasian	--	NS	--	--	2 studies (541/589) –0.17^**^	--	--
East Asian	NS	4 studies (540/991) 0.72^*^	--	--	2 studies (118/278) –0.07^*^	--	--
Cohort/Control	8 studies (4,047/5,557) 0.09^*^	NS	NS	NS	NS	**--**	5 studies (2,658/4,703) –0.06^**^
***Subgroups***							
European	NS	**--**	NS	3 studies (1,645/2,603) –0.08^*^	**--**	**--**	NS
Caucasian	5 studies (2,402/2,954) 0.17^****^	--	NS	NS	--	--	2 studies (1,015/1,206) –0.10^*^

Notes: ^*^p < 0.05, ^**^p < 0.01, ^***^p < 0.001, ^****^p < 0.0001; NS: Not significant; --: No data;

**Table 2:** Pooled meta-analysis: association of *MTHFR* 677 genotypes with homocysteine levels (mmol/L) and risk of colorectal cancer (2 studies)

Genotype (Number of studies)	Case *N* = 463 *n* (%) Mean ± SD (Range)	Control *N* = 470 *n* (%) Mean ± SD (Range)	Test of heterogeneity			Test of association	
			Q	*p*	*I*^*2*^ (%)	Pooled effect size (95% Cl)	*p*
***TT*** (2)	100 (21) 16.12 ± 1.07 (15.36–16.88)	72 (15) 15.67 ± 2.66 (13.79–17.56)	1.87	0.1705	46.8	0.31 (0.00–0.61)	0.0464
***CT*** (2)	207 (45) 12.69 ± 0.34 (12.45–12.94)	229 (49) 9.84 ± 0.19 (9.71–9.98)	2.43	0.1185	59	1.09 (0.89–1.29)	<0.0001
***CC*** (2)	156 (34) 11.74 ± 0.10 (11.67–11.82)	169 (36) 9.27 ± 0.37 (9.01–9.54)	0.39	0.5277	0	1.09 (0.86–1.33)	<0.0001

Notes: Q = Cochran’s Q; CI = Confidence interval.

**Table 3:** Schema of significant dietary parameters on the risks of colorectal cancer (CRC) and adenomas/polyps (AP) per case-control or cohort-control study designs and ethnic subgroups

Number of studies (n Case/n Control)	B12		Methionine	B9 (Folate)	B6	B2	Multivitamin	Fiber
	CRC	AP	CRC	CRC	CRC	CRC	CRC	CRC
Case/Control	8 studies (3,337/4,460) ES = 0.07^*^ 4 studies (2,125/2,728) RR, high level = 1.04^*^	NS	10 studies (3,014/4,156) ES: NS *3 studies (2,028/3,013)* RR, high level = 0.53^****^	**ES = NS** *9 studies (3,258/4,407)* RR, high level = 0.94^**^	*3 studies (3,075/4,137)* RR, high level = 0.96^**^	ES:NS	–	7 studies (5,564/7,417) ES = −0.09^*^
***Subgroups***								
R	ES:NS	–	2 studies (751/979) ES = 0.14^**^ Caucasian	ES:NS	–	2 studies (751/979) ES = 0.13^**^	–	–
CR	ES:NS	–	ES:NS	**ES = NS**	–	ES:NS	–	–
European	–	ES:NS	–	ES:NS RR:NS	–	–	–	
Caucasian	6 studies (2,998/3,965) ES = 0.07^*^ 3 studies (2,018/2,504) RR, high level = 1.04^*^	ES:NS	3 studies (2,028/3,013) RR, high level = 0.53^****^	ES = NS RR:NS	–	2 studies (751/979) ES = 0.13^**^	–	4 studies (3,197/4,200) ES = −0.08^***^
East Asian	–	–	–	3 studies (1,150/1,651) ES = −0.12^*^ 3 studies (1,150/1,651) RR, high level = 0.89^**^	–	–	–	–
Cohort/Control	–	–	NS	ES:NS *3 studies (933/1,564)* RR, high level = 0.92^*^	ES:NS	–	*7 studies (1,791/3,593)* RR, low level = 1.13^*^	–
***Subgroups***								
European	–	–	ES:NS	ES:NS RR:NS	2 studies (178/178) ES = −0.17^*^	–	–	–
Caucasian	–	–	–	4 studies (3,790/135,100) ES = −0.86^**^ 3 studies (933/1,564) RR, high level = 0.92^*^	–	–	6 studies (1,503/3,018) RR, high level = 0.81^*^	–
East Asian	–	–	2 studies (361/918) ES = 0.15^*^	ES:NS RR:NS	–	–	–	–

Notes: ES: Effect size; RR: Risk ratio; ^*^p < 0.05, ^**^p < 0.01, ^***^p < 0.001, ^****^p < 0.0001; NS: Not significant; --: No data; Blue font denotes relative risk (RR) related parameters.

### Homocysteine-related blood test measurements

#### Homocysteine levels

**Figure 2:**
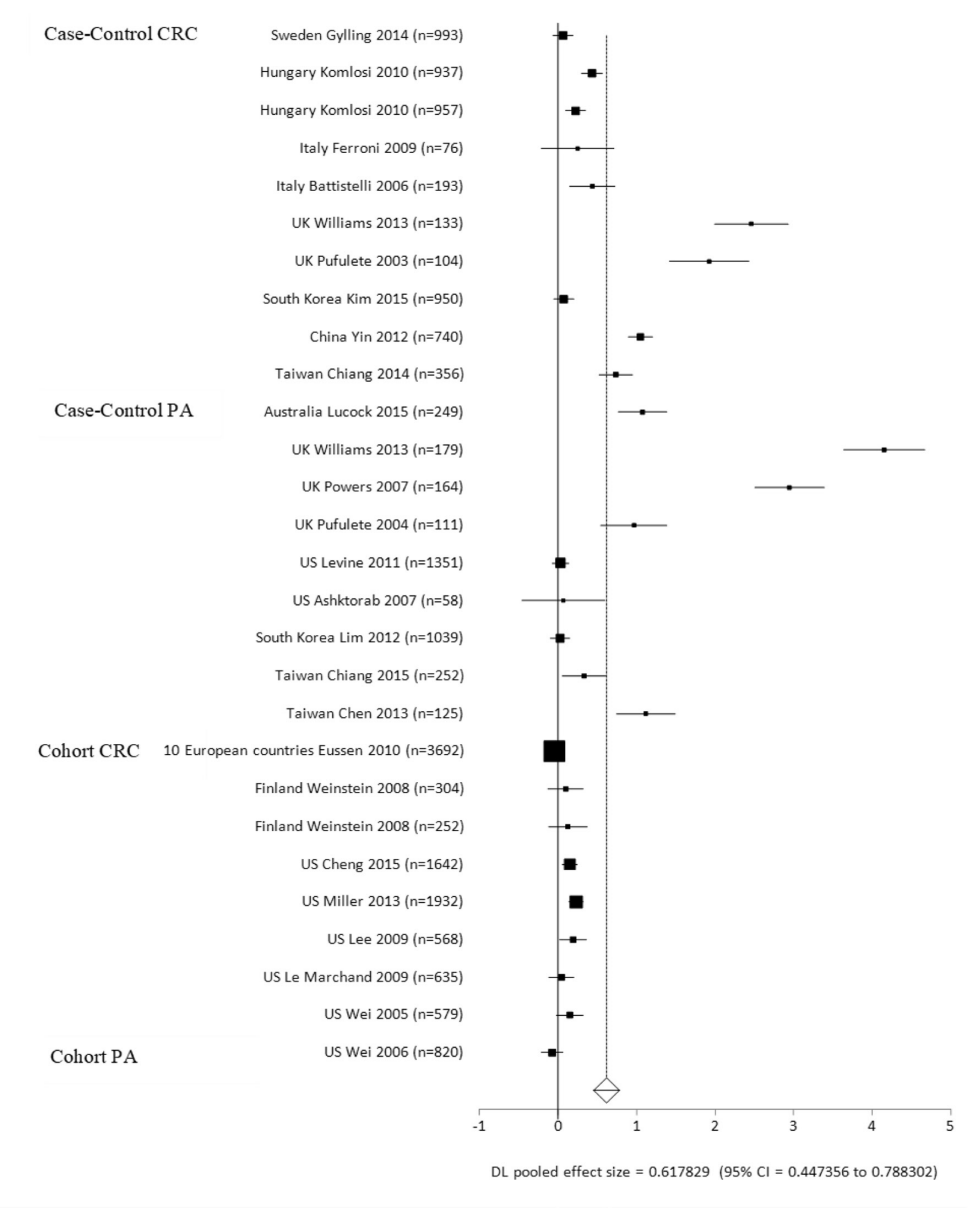
Forest plot for meta-analysis of homocysteine levels on the risks of colorectal cancer (CRC) and adenomatous polyps (AP) per case-control and cohort-control study designs

We present an overview of significant homocysteine-related measurements (from blood tests of plasma or serum) associated with the risk of CRC by ES (a positive ES value: increased risk; a negative ES value: decreased risk) in Table 1. A total of 37 studies were analyzed for these measurements, and pooled analyses was performed per study types (case-control or cohort), cancer or polyps case types (CRC, AP), cancer sites, and ethnic groups (Supplementary Table 2). Case-control and cohort studies combined had elevated homocysteine levels compared to the healthy controls, with a mean difference of 1.06 micromoles per liter (mmol/L) (ES = 0.62, *p <* 0.0001) (Supplementary Table 2; Figure 2, Forest Plot). Individually, in both case-control and cohort studies, cases had elevated homocysteine levels compared to the healthy controls, with mean differences of 1.43 mmol/L (ES = 0.92, *p* < 0.0001) for case-control, and 0.27 mmol/L (ES = 0.09, *p* = 0.0429) for the cohort studies. The case-control studies had a greater ES compared to the cohort studies. For the cancer sites in cohort studies and homocysteine levels, there were not enough studies per colon and rectal sites individually to see a difference between cases and controls. However, the ES was significant for homocysteine levels in CRC studies (ES = 0.62, *p* = 0.002) (Table 1).

For the case-control studies, CRC and AP individually had elevated homocysteine levels compared to the healthy control group, with mean differences of 1.43 mmol/L (ES = 0.71, *p* < 0.0001) for CRC group, and 1.13 mmol/L (ES = 1.13, *p* = 0.0001) for the AP group comparisons. The AP group had a greater ES than the CRC group in case-control studies. Amongst Europeans, homocysteine level was higher in the CRC and AP groups than the healthy control groups, with mean differences of 1.09 mmol/L (ES = 0.51, *p* < 0.0001) for CRC group, and 0.87 mmol/L (ES = 1.65, *p* = 0.0054) for the AP group when compared to the control groups. There were no significant differences in homocysteine levels for other subgroups including East Asian CRC and AP cases and Caucasian AP cases in comparison to the controls (Supplementary Table 2). Within the cohort studies, the CRC group had higher homocysteine levels than the control group, with a mean difference of 0.34 mmol/L (ES = 0.11, *p* = 0.0169). For Caucasians in the cohort studies, the CRC group had higher homocysteine levels compared to the control group, with a mean difference of 0.40 mmol/L (ES = 0.17, *p* < 0.0001). There was no significant difference in homocysteine levels for Europeans’ CRC group in comparison to the control group.

### Related blood test levels

**Figure 3:**
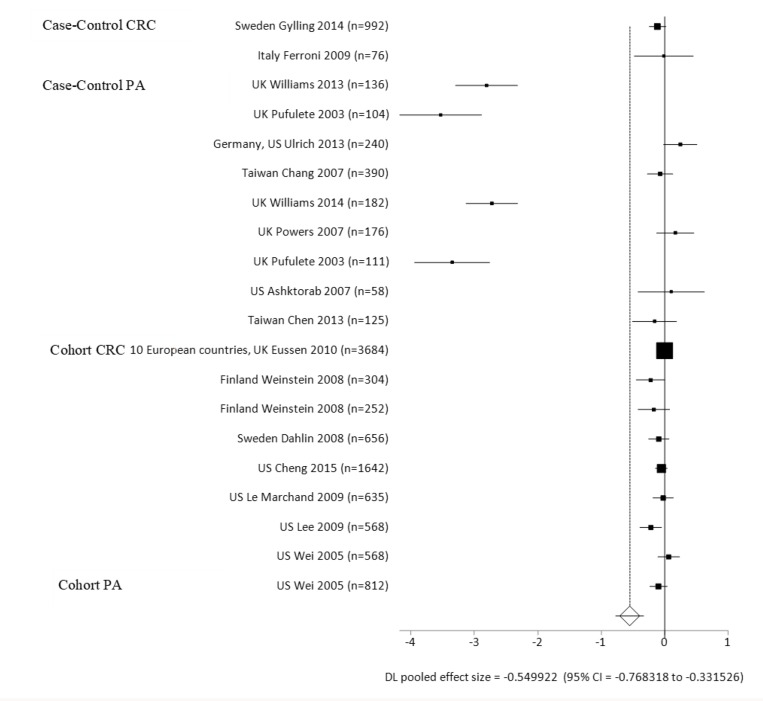
Forest plot for meta-analysis of B12 blood levels on the risks of colorectal cancer (CRC) and adenomas/polyps per case-control and cohort-control study designs

Case-control and cohort studies combined had lower B12 blood levels than the healthy control groups, with a mean difference of −11.46 pmol/L (ES = −0.55 *p* < 0.0001) (Supplementary Table 2; Figure 3, Forest Plot). Individually, the case-control and cohort groups had lower B12 blood levels than the healthy control group, with mean differences of –10.77 picomoles per liter (pmol/L) (ES = −1.08, *p* = 0.0009) for the case-control group, and –12.29 mmol/L (ES = −0.05, *p* = 0.0242) for the cohort-control group comparisons. For the case-control studies, the CRC group had lower B12 blood levels that the control group, with a mean difference of –5.83 pmol/L (ES = −0.99, *p* = 0.0086). European CRC cases had lower B12 blood levels when compared to the healthy controls, with a mean difference of –3.6 pmol/L (ES = −1.21, *p* = 0.0244). There was no significance in the AP cases or for the European subgroup on B12 levels when compared to the controls. Within the cohort studies, there were no significant differences in the European or Caucasian ethnic subgroups compared to the controls. For cancer sites and B12, there were not enough number of studies per colon and rectal sites individually to see a significant difference between the case and control groups.

There was no significance for folate levels in case-control or cohort studies combined. For the case-control studies, there were no significant differences in CRC or AP groups individually or per ethnic subgroup, except in the Caucasian and East Asian AP subgroups, both had lower folate blood levels than the control groups, with a mean difference of –1.25 nmol/L (ES = −0.17, *p* = 0.0038) for Caucasians. For the cohort studies, there was no difference in folate levels with CRC and AP individually, or per ethnic subgroup except in the European CRC group which had lower folate levels than the control, with a mean difference of –0.13 mmol/L (ES = −0.08, *p* = 0.0129).

The CRC group had lower methionine blood levels than the healthy control group, with a mean difference of –0.6 mmol/L (ES = −0.29, *p* = 0.0303) for 2 studies only conducted with Europeans using case-control designed studies. The CRC group had lower B6 levels than the healthy control group, with a mean difference of –3.38 nanomoles per liter (nmol/L) (ES = −0.06, *p* = 0.0070). In the Caucasian subgroup, CRC cases had lower B6 levels than the control group, with mean difference of –7.35 nmol/L (ES = −0.10, *p* = 0.0119). B2 Level was not significantly different between cases and controls across CRC and AP subgroups, which was conducted in the European population only (Supplementary Table 2).

### *MTHFR* C677T and homocysteine levels

**Figure 4:**
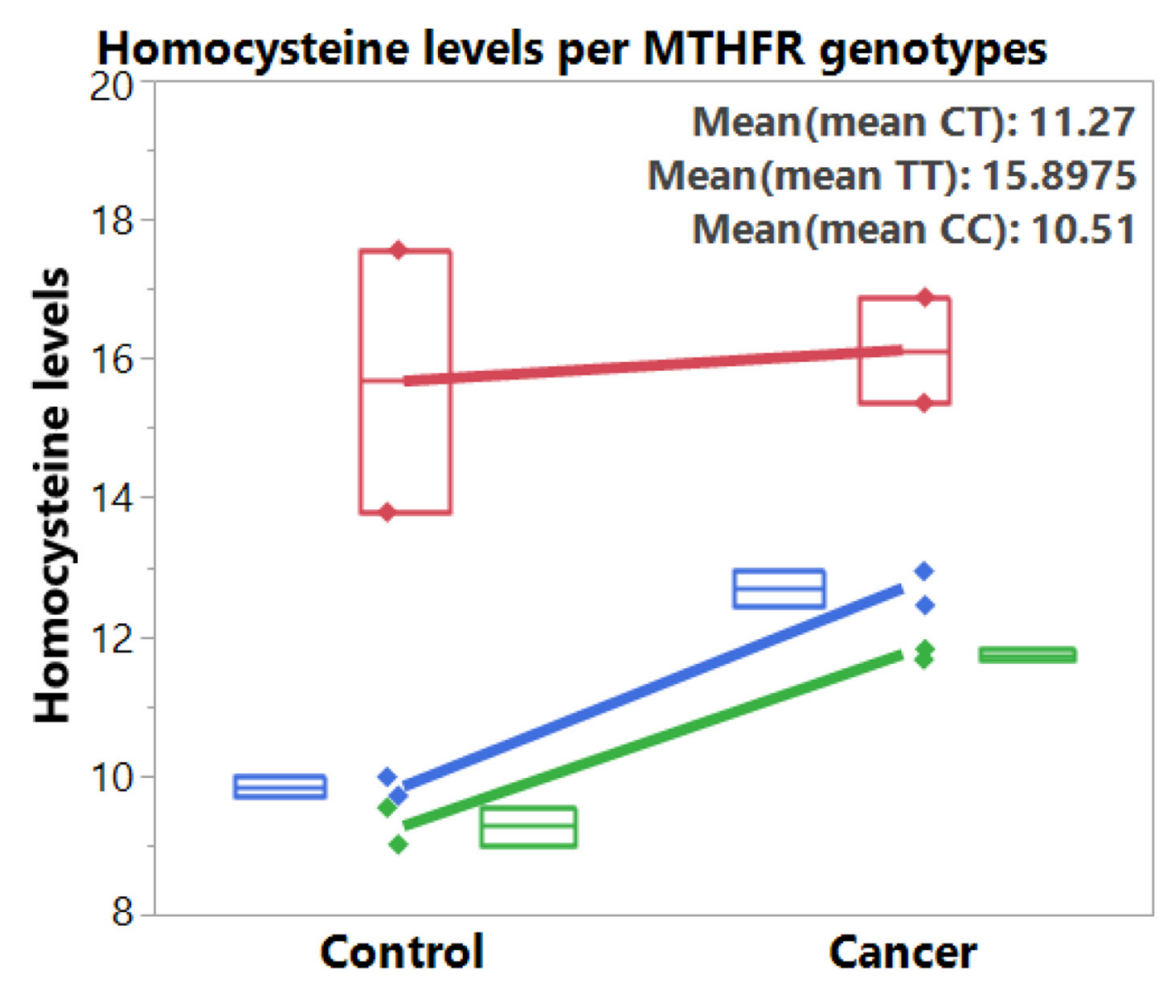
Box plots and fitted lines for homocysteine levels per *MTHFR* 677 genotypes (TT: red, CT: blue, CC: green) for control and colorectal cancer groups

From the two pooled studies [49, 50] with homocysteine and *MTHFR* data, we found higher homocysteine levels among CRC patients than in the control groups across the three genotypes (Table 2, Figure 4). *MTHFR* polymorphism was associated with increased homocysteine levels, with TT genotype having the highest homocysteine levels than the CT genotype and then the CC wildtype. The mean differences between the CRC and control group is 0.45 mmol/L (ES = 0.31, *p* = 0.0464) for TT genotype, 2.85 mmol/L (ES = 1.09, *p* < 0.0001) for CT genotype, and 2.47 mmol/L (ES = 1.09, *p* < 0.0001) for CC genotype.

### Dietary factors

An overview of significant dietary factors by ES based on means and SDs (a positive ES value: increased risk; a negative ES value: decreased risk), and RR based on frequency counts per groups (RR > 1: increased risk, versus RR < 1: decreased risk), are presented in Table 3. A total of 40 studies were analyzed for dietary factors based on data for ES (Supplementary Table 3A) and 42 studies based on data for RR analyses (Supplementary Table 3B). Overall, there were no significant differences on the results per case-control and cohort studies combined or separated in the analyses.

For the case-control studies, the CRC group had higher dietary B12 levels than the healthy control group, with a mean difference of 0.19 micrograms per day (mcg/d) (ES = 0.07, *p* = 0.0033). Caucasian CRC cases had higher dietary B12 levels than control group, with a mean difference of 0.25 mcg/d (ES = 0.07, *p* = 0.0041). There was no significant difference on dietary B12 intake levels among European and Caucasian AP cases in case-control studies, and among European CRC cases in cohort studies, compared to the control groups. Higher dietary B12 intakes (>3.5–7.8 mcg/d) increased CRC risk (RR = 1.04, *p* = 0.0424) and lower dietary B12 intake levels (< 3.5–4.8 mcg/d) was protective against CRC (RR = 0.91, *p* = 0.0436) in Caucasian (Supplementary Table 3B).

East Asian CRC cases had higher dietary methionine intakes than the healthy control group, with a mean difference of 0.08 milligrams per day (mg/d) (ES = 0.15, *p* = 0.015) (Supplementary Table 3A). Higher levels of methionine in Caucasian (≥1.4–2.5 grams per day (g/d)) was protective against CRC (RR = 0.53, *p* < 0.0001) (Supplementary Table 3B). There was no significant difference in dietary methionine intake levels for case-control and cohort studies combined, individually, or per ethnic subgroup in European and Caucasian CRC or AP groups compared to the control groups.

In case-control studies, East Asian CRC groups had lower dietary folate intakes than healthy controls, with a mean difference of –15.6 mcg/d (ES = −0.12, *p* = 0.0036) (Supplementary Table 3A). There was no significant difference on dietary folate intake levels in European or Caucasian CRC and AP groups compared to the controls in case-control studies. In cohort studies, the Caucasian CRC group had lower dietary folate intake than the control group, with a mean difference of −66.74 mcg/d (ES = −0.86, *p* = 0.0068). There was no significant difference on dietary folate levels in European and Caucasian CRC and Caucasian AP compared to the controls in cohort studies. High levels of dietary folate (>282.72–508 mcg/d) was protective against CRC (RR = 0.94, *p* = 0.0007) and low folate levels increased CRC risk (RR = 1.05, *p* = 0.0006) for case-control and cohort studies combined. Individually, high levels of folate in case-control studies (>282.72–508 mcg/d) was protective against CRC (ES = 0.94, *p* = 0.0015), as was high levels of folate in cohort studies (>375 mcg/d; ES = 0.94, *p* = 0.0491). For East Asian in case-control studies, higher levels (>282.72–484 mcg/d) of dietary folate were protective against CRC (RR = 0.89, *p* = 0.0072) and lower levels (<169.8–484 mcg/d) increased risk (RR = 1.09, *p* = 0.0064). For Caucasian in the cohort studies, higher levels of dietary folate (≥542 mcg/d) were protective against CRC risk (ES = 0.92, *p* = 0.0471) and lower levels (<242–542 mcg/d) increased risk (ES = 1.06, *p* = 0.0446). There was no significant difference on dietary folate intake levels between CRC and control groups for European or Caucasian in the case-control studies, or between AP and control groups for Caucasian in the cohort studies. There were also no significant differences between the cancer or polyp cases and control groups on folate supplement use across ethnic subgroups (Supplementary Table 2B).

For the case-control studies, the case group had lower B6 intake levels than the control group, with a mean difference of –0.27 mg/d (ES = −0.22, *p* = 0.0405). In cohort studies, there was no significant difference between the cases (CRC and AP) and controls, and across ethnic subgroups except for European CRC, which had lower mean dietary B6 than the healthy control group, with a mean difference of –0.11 mg/d (ES = −0.17, *p* = 0.0438) (Supplementary Table 3A). There was no significant difference on dietary or supplemental B6 intake levels for European, Caucasian and East Asian CRC cases compared to the control groups in both case-control and cohort studies.

Caucasian CRC cases in case-control studies had higher dietary B2 than the control group, with a mean difference of 0.15 mg/d (ES = 0.12, *p* = 0.0094) (Supplementary Table 3A). There was no significant difference in dietary B2 intake levels between case-control or cohort studies individually or combined compared to the control group. When analyzed per cancer site, dietary methionine (ES = 0.14, *p* = 0.003) and dietary vitamin B2 (ES = 0.13, *p* = 0.0094) levels were significantly different per rectum cases compared to the controls (Table 3).

The case group had lower levels of dietary fiber than the control when case-control and cohort studies were combined, compared to the control group, with a mean difference of –0.47 g/d (ES = −0.07, *p* = 0.0106). The case group in case-control studies had lower fiber intake levels compared to the control, with a mean difference of –0.52 g/d (ES = −0.09, *p* = 0.0134). There were no significant differences across ethnic subgroups between cancer and polyp cases and control, except for Caucasian in the CRC group which had taken lower levels of fiber than those in the control group, with a mean difference of –0.43 g/d (ES = −0.08, *p* = 0.0003) (Supplementary Table 3A). Within the cohort studies, there were no significant differences for cohort studies, CRC and AP, individually (Supplementary Table 3A) per cases for ethnic subgroups compared to the controls (Supplementary Table 3B). In cohort studies, those who did not take multivitamins were at increased risk for CRC (ES = 1.1, *p* = 0.0333), as were Caucasian who did not take multivitamins (ES = 1.17, *p* = .0135). There were no significant differences in cancer or polyp cases and controls per ethnic subgroups for case-control studies.

### Lifestyle factors

An overview of significant lifestyle factors by ES and RR is presented in Table 4. A total of 25 studies were analyzed for lifestyle factors based on data with ES (Supplementary Table 4A) and 61 studies based on data with RR analyses (Supplementary Table 4B) per case types, cancer sites and ethnic subgroups. For alcohol and smoking, there were not enough studies to find significant differences between colon, rectum, and CR combined (Supplementary Table 4A, 4B).

**Table 4:** Schema of significant lifestyle factors on the risks of colorectal cancer (CRC) and adenomas/polyps (AP) per case-control or cohort-control study designs and ethnic subgroups

Number of studies (n Case/n Control)	Alcohol		Smoking
	CRC	AP	AP
Case/Control	ES:NS RR:NS	8 studies (2,243/2,482) ES = 0.37^***^ 7 studies (1,926/2,097) RR, high level = 1.09^**^	2 studies (896/976) ES = 0.1^*^ 11 studies (2,314/3,171) RR, high level = 1.44^****^
***Subgroups***			
European	ES:NS 4 studies (230/425) RR, low level = 0.82^**^	ES:NS 2 studies (71/104) RR, low level = 0.73^*^	4 studies (955/1,220) RR, low level = 0.82^****^
Caucasian	ES:NS	3 studies (1,423/1,621) ES = 0.12^***^ 3 studies (704/739) RR, high level = 1.12^**^	2 studies (896/976) ES = 0.1^*^ 3 studies (839/1,033) RR, high level = 1.64^****^
East Asian	–	–	4 studies (520/918) RR, high level = 1.23^****^
Cohort/Control	ES:NS RR:NS	ES:NS RR:NS	5 studies (2,986/4,553) ES = 0.17^****^ 7 studies (2,217/4,197) RR, high level = 1.11^*^
***Subgroups***	–	–	
Caucasian	–	–	5 studies (2,986/4,553) ES = 0.17^****^ RR:NS

Notes: ES: Effect size; RR: Risk ratio; ^*^p < 0.05, ^**^p < 0.01, ^***^p < 0.001, ^****^p < 0.0001; NS: Not significant; –: No data; Blue font denotes relative risk related parameters.

Case-control and cohort studies combined had higher alcohol intake levels than the healthy control, with a mean difference of 2.86 g/d (ES = 0.1, *p* = 0.0013) (Supplementary Table 4A). When analyzed individually, the case group in the case-control studies had higher alcohol intake than the healthy control group, with a mean difference of 4.25 g/d (ES = 0.17, *p* = 0.0004). The AP group had higher alcohol intake than the control group, with a mean difference of 7.05 g/d (ES = 0.37, *p* = 0.0005). Caucasian AP cases also had higher alcohol intake levels than the control group, with a mean difference of 2.45 g/d (ES = 0.15, *p* < 0.0001) (Supplementary Table 4A). There was no significant RRs for Caucasian, or East and South Asian in relation to alcohol intake levels between CRC and control groups, except for the European CRC subgroup where lower levels of alcohol were protective for CRC risk (RR = 0.82, *p* = 0.0033). When analyzed per case type for RR in the case-control studies, lower alcohol levels were protective against AP (ES = 0.86, *p* = 0.0001), while higher levels (1–80 g/d) increased risk of AP (ES = 1.07, *p* = 0.0021). Lower alcohol intake levels were also protective for European (ES = 0.73, *p* = 0.026) and Caucasian (ES = 0.86, *p* = .0008). There was no significant difference in cancer or polyp cases (CRC or AP) compared to controls and across ethnic subgroups in cohort studies. (Supplementary Table 4B).

**Figure 5:**
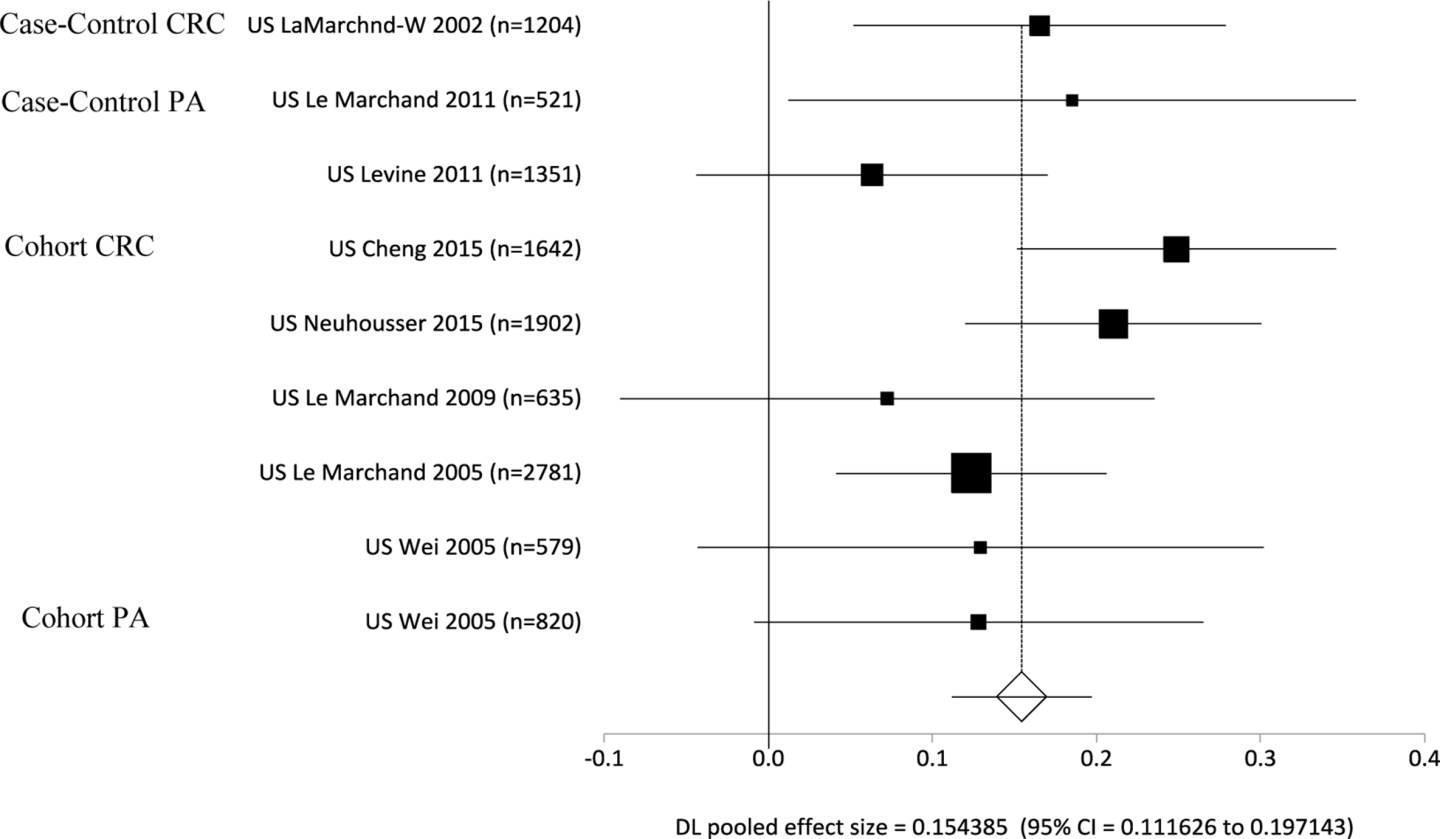
Forest plot for meta-analysis of smoking on the risks of colorectal cancer (CRC) and adenomas/polyps (AP) per case-control and cohort-control study designs

The case groups in case-control and cohort studies combined had higher number of packs smoked per year compared to the healthy controls, with a mean difference of 3.62 packs/year (ES = 0.16, *p* < 0.0001) (Supplementary Table 4A; Figure 5, Forest Plot). In case-control studies, higher levels of smoking were found in the case groups compared to the healthy control groups, with a mean difference of 4.66 packs/year (ES = 0.12, *p* = 0.0006) (Supplementary Table 4A). The AP group in case-control studies had higher packs smoked per year compared to the control group (ES = 0.1, *p* = 0.0364). The CRC group in cohort studies had higher packs smoked per year compared to the control group (ES = 0.17, *p* < 0.0001). Significance was found in the cases of case-control and cohort studies combined, where never or former smokers (RR = 0.97, *p* = 0.0105) were protected against CRC/AP and current smokers (RR = 1.09, *p* = 0.0031) had increased risk for CRC/AP. There was no significance in CRC and controls per ethnic subgroups for smoking within the case-control studies. When the AP group was analyzed individually, never or former smokers were protected against AP (ES = 0.86, *p* < 0.0001) as compared to current smokers which had increased risk for AP (ES = 1.44, *p* < 0.0001). Across ethnic subgroups, never or former smokers were protected against AP and currents smokers had increased risk. In European, never or former smokers had a protective effect for AP risk (RR = 0.91, *p* < 0.0001) compared to current smokers (ES = 1.64, *p* < 0.0001). In Caucasian, never and former smokers had a decreased risk of AP (RR = 0.79, *p* < 0.0001) compared to current smokers (RR = 1.64, *p* < 0.0001). In East Asian, never and former smokers were also protected against AP (RR = 0.79, *p* < 0.0001), compared to current smokers (RR = 1.23, *p* < 0.0001). Within the cohort studies, with CRC and AP combined, current smokers had increased risk for CRC/AP (ES = 1.14, *p* = 0.0013). When analyzed individually, current smokers had increased risk for CRC (ES = 1.11, *p* = 0.0119). There is no significance in cases (CRC or AP) compared to control for smoking status across ethnic subgroups in the cohort studies (Supplementary Table 4B).

## DISCUSSION

In this meta-analysis, we investigated homocysteine-related blood test measurements, and dietary and lifestyle factors involved in the OCM pathway for the risk of CRC. Higher homocysteine levels were associated with the increased risk of CRC, and *MTHFR* 677 gene polymorphisms were associated with increased homocysteine levels and CRC risks. Associated blood test measurements support the enzyme functions in homocysteine recycling to prevent the toxicity. Dietary and lifestyle factors contribute further to the compensatory mechanisms or mitigate the effects of toxicity from elevated homocysteine levels. Hence, our meta-analyses provided more definitive scientific evidence to suggest monitoring closely on homocysteine-related measurements for best practice on CRC prevention, potentially through the compensatory mechanisms in the OCM pathways for CRC prevention.

Our study highlights the role of homocysteine metabolism and related measurements in carcinogenesis for CRC, to provide a synthesis of current scientific evidence. Previous studies presented the associations of increased homocysteine levels with microsatellite instability (MSI) in CRC case-only design (no control group) [51], and MSI with *MTHFR* 677 TT genotype [52]. Both *MTHFR* 677 TT genotype and increased homocysteine levels can lead to methyl donor deficiency that can increase MSI particularly for aging populations [52]. Lower concentrations of nutrients related to the OCM pathway, such as folate, and B vitamins (B6, B12, B2) led to elevated homocysteine levels, which decreased OCM pathway activities for epigenetic mechanisms. Insufficient methyl groups in the diet and blood levels compromised DNA methylation, synthesis or repair, thus potentially promoted carcinogenesis [21, 46].

Folate may be supplemented to compensate for increased levels of homocysteine, for DNA synthesis and methylation to prevent carcinogenesis in early prevention [9, 49]. However, folate supplementation remains controversial for later stages of CRC for complexity in carcinogenesis in aggressive cancers. The findings from this meta-analysis presented that increased blood folate (B9) levels were associated with decreased risk of CRC, in European subgroup with a small ES (−0.08) conducted using case/control study design. For dietary measurements, increased dietary folate levels was also protective against CRC using risk ratio (0.94 being < 1) for all populations combined, and in East Asian (RR of 0.89) conducted using case/control study design and in Caucasian (RR of 0.92) conducted using cohort/control study design. In Caucasian subgroup using the cohort/control study design, the ES was larger (−0.89) than ES (−0.12) conducted using the case/control study design in East Asian subgroups. The differences of effects per ES and RR calculations were complex, with various distribution patterns across world’s regions. To summarize across the studies, folate intake level of >282 mcg/day was presented across the studies as the minimum beneficial level worldwide. Future studies can continue to examine the minimum beneficial levels of various dietary methyl donors for CRC prevention. The AICR published 10 recommendations to lower CRC risk, including reducing alcohol intake, and reducing red meat intake [28]. Along with these recommendations, being mindful of calorie intake and physical activity, and enjoying a plant-based diet that is rich in dietary fiber to supply folate were advised to prevent CRC [49].

For vitamin B12 and methionine, our analyses presented opposite levels in the blood and dietary measurements. For B12 blood levels, CRC and AP groups had lower levels than the healthy counterparts; whereas, CRC and AP groups had higher dietary B12 intake levels than the control groups. For methionine, the CRC group had lower blood levels than the healthy control group; whereas, the CRC group had higher dietary B12 intake levels than the control group. The differences could be due to multiple reasons. Most plausible reason being that while CRC patients consume higher B12 and methionine levels then the control groups, their B12 and methionine blood levels remain lower than the control groups possibly due to wasting to the cancer cells. Future studies are needed to follow these blood tests and dietary measurements longitudinally to observe chronological changes on the higher consumption of these nutrients and the need for more supplementation of these two nutrients to the CRC cases. Deficiency of these nutrients in blood levels may be more important to follow as the basis for supplementation in meeting the needs for the OCM pathways [6–10].

Identifying specific dietary factors linked with CRC risk is challenging because of the complex composition of food and the fact that dietary changes will affect multiple nutrients [50]. Given the limited number of studies for some factors and in certain regions and racial-ethnic groups from this meta-analysis study, further studies are warranted to clarify and provide more substantive evidence for added evidences. Before general advice can be given, it is important to understand how a person’s nutritional requirement is dependent on one’s genetic profile, as well as the different components of foods and vitamins one is taking and the blood levels. For example, while protein-based food can supply B12 and methionine, the way red meat is processed or cooked can affect not only the dietary intake levels, but also inflammatory process associated with the fatty acids which might increase the CRC risk [24, 25]. Total fiber can be further broken down to insoluble fiber found in foods such as cereals or soluble fiber found in foods such as fruits and vegetables [53–56]. In a population of Swedish women, higher fruit intake was associated with a reduction in CRC risk, while higher intake of cereal fiber did not lower CRC risk [55]. Further studies can be conducted to examine further on the fiber types in various foods and their risks associated with CRC [57–60].

## MATERIALS AND METHODS

### Study search strategy

Following the guidelines for reporting meta-analyses of observational studies [61], we searched the online databases of PubMed, PubMed Central, Cochrane databases, Embase, Google Scholar and Airiti Library to identify and access all available studies, from 1995 (year in which the first related study was published) to September 2017. We used the search terms colon cancer, rectal cancer, colorectal cancer, *MTHFR* gene, *MTHFR* in CRC, epigenetics, nutrigenetics, environment, diet, diet prevention, folate, folate pathways, blood and/or plasma folate, homocysteine, micronutrients, dietary fiber, red meat, iron, lifestyle, behavior, case–control design and meta-analysis, then entered the resulting articles into a database organized by key words. We used previous meta-analysis and review papers to cross check and trace back to all original studies. Two raters, one who was familiar with the literature search process and organization and one who was familiar with meta-analytic methods, conducted the literature search at four different times at least 3 months apart until all possible studies were identified.

### Inclusion/exclusion criteria

The inclusion criteria were: the studies: 1) included data for the association of homocysteine-related blood tests, and dietary and lifestyle factors with CRC risk using a case-control, cohort or RCT design, 2) were written in English, or 3) were written in non-English but provided tables that clearly listed blood, dietary or lifestyle measurement levels or counts. Articles with *MTHFR* genotype frequency counts in association with homocysteine and related factors and CRC risk were also identified and analyzed separately. Articles were excluded if they 1) were not written in English and without tables listing any counts or statistical measurements, 2) did not provide data per case or cohort versus control groups or 3) had duplicate use of data by another study with more comprehensive data.

Of the 301 articles we identified involving CRC and homocysteine related factors, we excluded 147 from the analysis because they did not provide data per case and control groups, including 6 meta-analyses. From the remaining 154 articles, we excluded 55 for not having any homocysteine-related blood tests, or dietary or lifestyle factors associated with colorectal health. Of the remaining 99 studies, 13 involved subsidiary or redundant use of data contained in other included studies that had more current and/or complete data, thus we excluded them as well. Finally, we included 86 articles with usable data for pooled analysis, 63 papers reported CRC only, 5 papers had both CRC and AP, and 18 reported AP disease only (Figure 1).

### Quality assessment

We evaluated each study for quality using a set of indicators appropriate for the current state of science for the field, integrated from multiple sources. The sources for these criteria included the Preferred Reporting Items for Systematic reviews and Meta-Analyses (PRISMA) guideline [62], guidelines on quality reporting for observational studies [63, 64], and the quality evidence from a previous meta-analysis of *MTHFR* and CRC [6, 7, 9, 21]. The details of the quality indicators that we used to assess the studies included in the meta-analysis are presented in Supplementary Table 1A. The total quality score could range from 0 to 30. Three sub scores were combined to obtain the total score: (1) external validity, with 10 items on demographic data (score range of 0–10); (2) internal validity, with 10 items on research methods and procedures (score range of 0–10); and (3) quality of reporting, with 10 items on the data and study results (score range of 0–10). Within the demographic data, CRC and AP were diagnosed by histology, pathology, review of medical records, both histology and pathology, or all three methods (see Diagnosis categories for each study in Supplementary Table 1A). Within the internal validity scoring, DNA analysis was an item counted for in four papers with *MTHFR* 677 genotype counts (*MTHFR* genotyping was determined using either polymerase chain reaction or matrix-assisted laser desorption/ionization methods) (Supplementary Table 1B), in association with homocysteine levels (Table 2).

The ranges of quality scores for all included studies were from 15 to 23. The reviewed studies thus all scored at 50% or higher of the total possible score of 0 to 30, suggesting that their findings are trustworthy. All studies included the use of biological samples of blood levels, and some form of a food questionnaire (ie. Food Frequency Questionnaire) for dietary and lifestyle levels. We checked data extractions and entry for accuracy and ran the preliminary analyses to make sure the ranges of entries and pooled results were accurate for all studies. The extracted data (mean, standard deviation, ranges, and counts) were all converted to the International System (SI) of Units and referenced using the recommended dietary allowance (RDA) by the National Institute of Health [65, 66]. We calculated the standard deviation for studies missing this data, from the range of confidence interval [67–69]. ES calculation was based on mean and standard deviation, and RR calculation based on frequency counts between case and control groups.

### Data synthesis and analysis

We entered data into Excel (Microsoft Corp, Redmond, WA), and analyzed data using StatsDirect Version 3 updated software (Cheshire, UK). We calculated pooled RR and 95% confidence intervals (CI) for the measurements between cases and controls for the associations with CRC or AP risks. We compared the pooled RRs to be more conservative and yielding standardized risk ratios per AICR guidelines [28]. We used JMP^®^ pro 13 programs (SAS Institute, Cary, NC, USA, 2015) for exploration on meta-predictive analyses, plots and curve-fitting of *MTHFR* genotypes and homocysteine levels.

## SUPPLEMENTARY MATERIALS TABLES
















