# Supplementary material for: Meta-analysis of homocysteine-related factors on the risk of colorectal cancer
Source: Oncotarget. 2018 May 22;9(39):25681–97. doi: 10.18632/oncotarget.25355 (PMC5986656; doi:10.18632/oncotarget.25355)
Supplement: Supplementary file 1 [file oncotarget-09-25681-s001.pdf]

Meta-analysis of homocysteine-related factors on the risk of colorectal cancer

SUPPLEMENTARY MATERIALS

REFERENCES

1. Fan Y, Jin X, Man C, Gao Z, Wang X. Meta-analysis of the association between the inflammatory potential of diet and colorectal cancer risk. Oncotarget. 2017; 8:59592–59600. https://doi.org/10.18632/oncotarget.19233.
2. Heine-Bröring RC, Winkels RM, Renkema JM, Kragt L, van Orten-Luiten AC, Tigchelaar EF, Chan DS, Norat T, Kampman E. Dietary supplement use and colorectal cancer risk: a systematic review and meta-analyses of prospective cohort studies. Int J Cancer. 2015; 136:2388–2401. https://doi.org/10.1002/ijc.29277.
3. Zhang D, Wen X, Wu W, Guo Y, Cui W. Elevated Homocysteine Level and Folate Deficiency Associated with Increased Overall Risk of Carcinogenesis: Meta-Analysis of 83 Case-Control Studies Involving 35,758 Individuals. PLoS One. 2015; 10:e0123423.
4. Kennedy DA, Stern, SJ, Matok I, Moretti ME, Sarkar M, Adams-Webber T, Koren G. Folate intake, MTHFR polymorphisms, and the risk of colorectal cancer: A systematic review and meta-analysis. J Cancer Epidemiol. 2012; 2012:952508. https://doi.org/10.1155/2012/952508.
5. Zacho J, Yazdanyar S, Bojesen SE, Tybjærg-Hansen A, Nordestgaard BG. Hyperhomocysteinemia, methylenetetrahydrofolate reductase c.677C>T polymorphism and risk of cancer: cross-sectional and prospective studies and meta-analyses of 75,000 cases and 93,000 controls. Int J Cancer. 2012; 128:644–652. https://doi.org/10.1002/ijc.25375.
6. Wakai K, Date C, Fukui M, Tamakoshi K, Watanabe Y, Hayakawa N, Kojima M, Kawado M, Suzuki K, Hashimoto S, Tokudome S, Ozasa K, Suzuki S, et al. JACC Study Group. Dietary fiber and risk of colorectal cancer in the Japan collaborative cohort study. Cancer Epidemiol Biomarkers Prev. 2007; 16:668–675.
7. Choi JH, Yates Z, Martin C, Boyd L, Ng X, Skinner V, Wai R, Veysey M, Lucock M. Gene-Nutrient Interaction between Folate and Dihydrofolate Reductase in Risk for Adenomatous Polyp Occurrence: A Preliminary Report. J Nutr Sci Vitaminol (Tokyo). 2015; 61:455–459. https://doi.org/10.3177/jnsv.61.455.
8. Joo Jeon Y, Woo Kim J, Mi Park H, Kim JO, Geun Jang H, Oh J, Gyu Hwang S, Won Kwon S, Oh D, Keun Kim N. Genetic variants in 3'-UTRs of methylenetetrahydrofolate reductase (MTHFR) predict colorectal cancer susceptibility in Koreans. Sci Rep. 2015; 5:11006. https://doi.org/10.1038/srep11006.
9. Levine AJ, Figueiredo JC, Lee W, Conti DV, Kennedy K, Duggan DJ, Poynter JN, Campbell PT, Newcomb P, Martinez ME, Hopper JL, Le Marchand L, Baron JA, et al. A Candidate Gene Study of Folate-Associated One Carbon Metabolism Genes and Colorectal Cancer Risk. Cancer Epidemiol Biomarkers Prev. 2010; 19:1812–1821. https://doi.org/10.1158/1055-9965.EPI-10-0151.
10. Eklöf V, Van Guelpen B, Hultdin J, Johansson I, Hallmans G, Palmqvist R. The reduced folate carrier (RFC1) 80G > A and folate hydrolase 1 (FOLH1) 1561C > T polymorphisms and the risk of colorectal cancer: a nested case-referent study. Scand J Clin Lab Invest. 2008; 68:393–401. https://doi.org/10.1080/00365510701805431.
11. Mohebbi SR, Khatami F, Ghiasi S, Derakhshan F, Atarian H, Zali MR. Reverse association between MTHFR polymorphism (C677T) with sporadic colorectal cancer. Gastroenterology and Hepatology From Bed to Bench. 2008; 1:57–63. https://doi.org/10.22037/ghfbb.v1i2.19.
12. Mokarram P, Naghibalhossaini F, Saberi Firoozi M, Hosseini SV, Izadpanah A, Salahi H, Malek-Hosseini SA, Talei A, Mojallal M. Methylenetetrahydrofolate reductase C677T genotype affects promoter methylation of tumor-specific genes in sporadic colorectal cancer through an interaction with folate/vitamin B12 status. World J Gastroenterol. 2008; 14:3662–3671.
13. Van Guelpen B, Hultdin J, Johansson I, Hallmans G, Stenling R, Riboli E, Winkvist A, Palmqvist R. Low folate levels may protect against colorectal cancer. Gut. 2006; 55:1461–1466. https://doi.org/10.1136/gut.2005.085480.
14. Hirose M, Kono S, Tabata S, Ogawa S, Yamaguchi K, Mineshita M, Hagiwara T, Yin G, Lee KY, Tsuji A, Ikeda N. Genetic polymorphisms of methylenetetrahydrofolate

reductase and aldehyde dehydrogenase 2, alcohol use and risk of colorectal adenomas: Self-Defense Forces Health Study. Cancer Sci. 2005; 96:513–518. https://doi.org/10.1111/j.1349-7006.2005.00077.x.

15. Kim DH, Ahn YO, Lee BH, Tsuji E, Kiyohara C, Kono S. Methylenetetrahydrofolate reductase polymorphism, alcohol intake, and risks of colon and rectal cancers in Korea. Cancer Lett. 2004; 216:199–205. https://doi.org/10.1016/j.canlet.2004.08.014.

16. Ulrich CM, Kampman E, Bigler J, Schwartz SM, Chen C, Bostick R, Fosdick L, Beresford SA, Yasui Y, Potter JD. Lack of association between the C677T MTHFR polymorphism and colorectal hyperplastic polyps. Cancer Epidemiol Biomarkers Prev. 2000; 9:427–433.

17. Slattery ML, Edwards SL, Samowitz W, Potter J. Associations between family history of cancer and genes coding for metabolizing enzymes (United States). Cancer Causes Control. 2000; 11:799–803.

18. Ma J, Stampfer MJ, Christensen B, Giovannucci E, Hunter DJ, Chen J, Willett WC, Selhub J, Hennekens CH, Gravel R, Rozen R. A polymorphism of the methionine synthase gene: association with plasma folate, vitamin B12, homocyst(e)ine, and colorectal cancer risk. Cancer Epidemiol Biomarkers Prev. 1999; 8:825–829.

19. Slattery ML, Potter JD, Samowitz W, Schaffer D, Leppert M. Methylenetetrahydrofolate reductase, diet, and risk of colon cancer. Cancer Epidemiol Biomarkers Prev. 1999; 8:513–518.

20. Nitter M, Norgård B, de Vogel S, Eussen SJ, Meyer K, Ulvik A, Ueland PM, Nygård O, Vollset SE, Bjørge T, Tjønneland A, Hansen L, Boutron-Ruault M, et al. Plasma methionine, choline, betaine, and dimethylglycine in relation to colorectal cancer risk in the European Prospective Investigation into Cancer and Nutrition (EPIC). Ann Oncol. 2014; 25:1609–15. https://doi.org/10.1093/annonc/mdu185.

21. Eussen SJ, Vollset SE, Hustad S, Midttun Ø, Meyer K, Fredriksen A, Ueland PM, Jenab M, Slimani N, Boffetta P, Overvad K, Thorlacius-Ussing O, Tjønneland A, et al. Plasma vitamins B2, B6, B12, and related genetic variants as predictors of colorectal cancer risk. Cancer Epidemiol Biomarkers Prev. 2010; 19:2549–2561. https://doi.org/10.1158/1055-9965.EPI-10-0407.

22. Eussen SJ, Vollset SE, Igland J, Meyer K, Fredriksen A, Ueland PM, Jenab M, Slimani N, Boffetta P, Overvad K, Tjønneland A, Olsen A, Clavel-Chapelon F, et al. Plasma folate, related genetic variants and colorectal cancer risk in EPIC. Epidemiol Biomarkers Prev. 2010; 19:1328–1340. https://doi.org/10.1158/1055-9965.EPI-09-0841.

23. Iacopetta B, Heyworth J, Girschik J, Grieu F, Clayforth C, Fritschi L. The MTHFR C677T and DeltaDNMT3B C-149T polymorphisms confer different risks for right- and left-sided colorectal cancer. Int J Cancer. 2009; 125:84–90. https://doi.org/10.1002/ijc.24324.

24. Weinstein SJ, Albanes D, Selhub J, Graubard B, Lim U, Taylor PR, Virtamo J, Stolzenberg-Solomon R. One-carbon metabolism biomarkers and risk of colon and rectal cancers. Cancer Epidemiol Biomarkers Prev. 2008; 17:3233–3240. https://doi.org/10.1158/1055-9965.EPI-08-0459.

25. Osian G, Procopciuc L, Vlad L. MTHFR polymorphisms as prognostic factors in sporadic colorectal cancer. J Gastrointestin Liver Dis. 2007; 16:251–256.

26. Komlósi V, Hitre E, Pap E, Adleff V, Réti A, Székely E, Bíró A, Rudnai P, Schoket B, Müller J, Tóth B, Ottó S, Kásler M, et al. SHMT1 1420 and MTHFR 677 variants are associated with rectal but not colon cancer. BMC Cancer. 2010; 10:525. https://doi.org/10.1186/1471-2407-10-525.

27. Pardini B, Kumar R, Naccarati A, Prasad RB, Forsti A, Polakova V, Vodickova L, Novotny J, Hemminki K, Vodicka P. MTHFR and MTRR genotype and haplotype analysis and colorectal cancer susceptibility in a case-control study from the Czech Republic. Mutat Res. 2011; 721:74–80. https://doi.org/10.1016/j.mrgentox.2010.12.008.

28. Myte R Gylling B, Schneede J, Ueland PM, Häggström J, Hultdin J, Hallmans G, Johansson I, Palmqvist R, Van Guelpen B. Components of One-carbon Metabolism other than Folate and Colorectal Cancer Risk. Epidemiology. 2016; 27:787–796. https://doi.org/10.1097/EDE.0000000000000529.

29. Gylling B, Van Guelpen B, Schneede J, Hultdin J, Ueland PM, Hallmans G, Johansson I, Palmqvist R. Low folate levels are associated with reduced risk of colorectal cancer in a population with low folate status. Cancer Epidemiol Biomarkers Prev. 2014; 23:2136–2144. https://doi.org/10.1158/1055-9965.EPI-13-1352.

30. Dahlin AM, Van Guelpen B, Hultdin J, Johansson I, Hallmans G, Palmqvist R. Plasma vitamin B12 concentrations and the risk of colorectal cancer: a nested case-referent study. Int J Cancer. 2008; 122:2057–2061. https://doi.org/10.1002/ijc.23299.

31. Ulvik A, Vollset SE, Hansen S, Gislefoss R, Jellum E, Ueland PM. Colorectal cancer and the methylenetetrahydrofolate reductase 677C ≥ T and methionine synthase 2756A ≥ G polymorphisms: a study of 2,168 case-control pairs from the JANUS cohort. Cancer Epidemiol Biomarkers Prev. 2004; 13:2175–2180.

32. Heijmans BT, Boer JM, Suchiman HE, Cornelisse CJ, Westendorp RG, Kromhout D, Feskens EJ, Slagboom PE. A common variant of the methylenetetrahydrofolate reductase gene (1p36) is associated with an increased risk of cancer. Cancer Res. 2003; 63:1249–1253.

33. Vossen CY, Hoffmeister M, Chang-Claude JC, Rosendaal FR, Brenner H. Clotting factor gene polymorphisms and colorectal cancer risk. J Clin Oncol. 2011; 29:1722–1727. https://doi.org/10.1200/JCO.2010.31.8873.

34. Ferroni P, Palmirotta R, Martini F, Riondino S, Savonarola A, Spila A, Ciatti F, Sini V, Mariotti S, Del Monte G, Roselli M, Guadagni F. Determinants of homocysteine levels in colorectal and breast cancer patients. Anticancer Res. 2009; 29:4131–4138.

35. Battistelli S, Vittoria A, Stefanoni M, Bing C, Roviello F. Total plasma homocysteine and methylenetetrahydrofolate reductase C677T polymorphism in patients with colorectal carcinoma. World J Gastroenterol. 2006; 12:6128–6132.

36. Theodoratou E, Farrington SM, Tenesa A, McNeill G, Cetnarskyj R, Barnetson RA, Porteous ME, Dunlop MG,

Campbell H. Dietary vitamin B6 intake and the risk of colorectal cancer. Cancer Epidemiol Biomarkers Prev. 2008; 17:171–182.

37. Sharp L, Little J, Brockton NT, Cotton SC, Masson LF, Haites NE, Cassidy J. Polymorphisms in the methylenetetrahydrofolate reductase (MTHFR) gene, intakes of folate and related B vitamins and colorectal cancer: a case-control study in a population with relatively low folate intake. Br J Nutr. 2008; 99:379–389.
38. Guerreiro CS, Carmona B, Gonçalves S, Carolino E, Fidalgo P, Brito M, Leitão CN, Cravo M. Risk of colorectal cancer associated with the C677T polymorphism in 5,10-methylenetetrahydrofolate reductase in Portuguese patients depends on the intake of methyl-donor nutrients. Am J Clin Nutr. 2008; 88:1413–8.
39. Ulrich CM, Toriola AT, Siegel EM, Brenner H, Chang-Claude J, Abbenhardt C, Kotzmann J, Song X, Owen RW, Hoffmeister M, Becher H, Shibata D, Vickers K, et al. Plasma 25-hydroxyvitamin D3, folate and vitamin B12 biomarkers among international colorectal cancer patients: a pilot study. J Nutr Sci. 2013; 2:e9. https://doi.org/10.1017/jns.2012.28.
40. Levine AJ, Figueiredo JC, Lee W, Poynter JN, Conti D, Duggan DJ, Campbell PT, Newcomb P, Martinez ME, Hopper JL, Le Marchand L, Baron JA, Limburg PJ, et al. Genetic variability in the MTHFR gene and colorectal cancer risk using the colorectal cancer family registry. Cancer Epidemiol Biomarkers Prev. 2010; 19:89–100. https://doi.org/10.1158/1055-9965.EPI-09-0727.
41. Sun Z, Liu L, Wang PP, Roebothan B, Zhao J, Dicks E, Cotterchio M, Buehler S, Campbell PT, McLaughlin JR, Parfrey PS. Association of total energy intake and macronutrient consumption with colorectal cancer risk: results from a large population-based case-control study in Newfoundland and Labrador and Ontario, Canada. Nutr J. 2012; 11:18. https://doi.org/10.1186/1475-2891-11-18.
42. Navarro SL, Neuhouser ML, Cheng TD, Tinker LF, Shikany JM, Snetselaar L, Martinez JA, Kato I, Beresford SA, Chapkin RS, Lampe JW. The Interaction between Dietary Fiber and Fat and Risk of Colorectal Cancer in the Women's Health Initiative. Nutrients. 2016; 8:E779. https://doi.org/10.3390/nu8120779.
43. Cheng TY, Makar KW, Neuhouser ML, Miller JW, Song X, Brown EC, Beresford SA, Zheng Y, Poole EM, Galbraith RL, Duggan DJ, Habermann N, Bailey LB, et al. Folate-mediated one-carbon metabolism genes and interactions with nutritional factors on colorectal cancer risk: Women's Health Initiative Observational Study. Cancer. 2015; 121:3684–3691. https://doi.org/10.1002/cncr.29465.
44. Cho E, Zhang X, Townsend MK, Selhub J, Paul L, Rosner B, Fuchs CS, Willett WC, Giovannucci EL. Unmetabolized Folic Acid in Prediagnostic Plasma and the Risk of Colorectal Cancer. J Natl Cancer Inst. 2015; 107:260. https://doi.org/10.1093/jnci/djv260.
45. Neuhouser ML, Cheng TY, Beresford SA, Brown E, Song X, Miller JW, Zheng Y, Thomson CA, Shikany JM, Vitolins MZ, Rohan T, Green R, Ulrich CM. Red blood cell folate and plasma folate are not associated with risk of incident colorectal cancer in the Women's Health Initiative observational study. Int J Cancer. 2015; 137:930–939. https://doi.org/10.1002/ijc.29453.
46. Ashmore JH, Lesko SM, Muscat JE, Gallagher CJ, Berg AS, Miller PE, Hartman TJ, Lazarus P. Association of dietary and supplemental folate intake and polymorphisms in three FOCM pathway genes with colorectal cancer in a population-based case-control study. Genes Chromosomes Cancer. 2013; 52:945–953. https://doi.org/10.1002/gcc.22089.
47. Miller JW, Beresford SA, Neuhouser ML, Cheng TY, Song X, Brown EC, Zheng Y, Rodriguez B, Green R, Ulrich CM. Homocysteine, cysteine, and risk of incident colorectal cancer in the Women's Health Initiative observational cohort. Am J Clin Nutr. 2013; 97:827–834. https://doi.org/10.3945/ajcn.112.049932.
48. Zschäbitz S, Cheng TY, Neuhouser ML, Zheng Y, Ray RM, Miller JW, Song X, Maneval DR, Beresford SA, Lane D, Shikany JM, Ulrich CM. B vitamin intakes and incidence of colorectal cancer: results from the Women's Health Initiative Observational Study cohort. Am J Clin Nutr. 2013; 97:332–343. https://doi.org/10.3945/ajcn.112.034736.
49. Curtin K, Samowitz WS, Ulrich CM, Wolff RK, Herrick JS, Caan BJ, Slattery ML. Nutrients in folate-mediated, one-carbon metabolism and risk of rectal tumors in men and women. Nutr Cancer. 2011; 63:357–366. https://doi.org/10.1080/01635581.2011.535965.
50. Le Marchand L, White KK, Nomura AM, Wilkens LR, Selhub JS, Tiirikainen M, Goodman MT, Murphy SP, Henderson BE, Kolonel LN. Plasma Levels of B Vitamins and Colorectal Cancer Risk: The Multiethnic Cohort Study. Cancer Epidemiol Biomarkers Prev. 2009; 18:2195–2201. https://doi.org/10.1158/1055-9965.EPI-09-0141.
51. Lee JE, Li H, Giovannucci E, Lee I, Selhub J, Stampfer M, Ma J. Prospective Study of Plasma Vitamin B6 and Risk of Colorectal Cancer in Men. Cancer Epidemiol Biomarkers Prev. 2009; 18:1197–1202. https://doi.org/10.1158/1055-9965.EPI-08-1001.
52. Murtaugh MA, Curtin K, Sweeney C, Wolff RK, Holubkov R, Caan BJ, Slattery ML. Dietary intake of folate and co-factors in folate metabolism, MTHFR polymorphisms, and reduced rectal cancer. Cancer Causes Control. 2007; 18:153–163. https://doi.org/10.1007/s10552-006-0099-2.
53. Koushik A, Kraft P, Fuchs CS, Hankinson SE, Willett WC, Giovannucci EL, Hunter DJ. Nonsynonymous polymorphisms in genes in the one-carbon metabolism pathway and associations with colorectal cancer. Cancer Epidemiol Biomarkers Prev. 2006; 15:2408–2417. https://doi.org/10.1158/1055-9965.EPI-06-0624.
54. Le Marchand L, Wilkens LR, Kolonel LN, Henderson BE. The MTHFR C677T polymorphism and colorectal cancer: the multiethnic cohort study. Cancer Epidemiol Biomarkers Prev. 2005; 14:1198–1203. https://doi.org/10.1158/1055-9965.EPI-04-0840.
55. Curtin K, Bigler J, Slattery ML, Cann B, Potter JD, Ulrich CM. MTHFR C677T and A1298C polymorphisms: diet, estrogen, and risk of colon cancer. Cancer Epidemiology, Biomarkers & Prevention. 2004; 13:285–292.

56. Keku T, Millikan R, Worley K, Winkel S, Eaton A, Biscocho L, Martin C, Sandler R. 5,10-methylenetetrahydrofolate reductase codon 677 and 1298 polymorphisms and colon cancer in African Americans and Whites. Cancer Epidemiology, Biomarkers & Prevention. 2002; 11:1611–1621.

57. Le Marchand L, Donlon T, Hankin JH, Kolonel LN, Wilkens LR, Seifried A. B-vitamin intake, metabolic genes, and colorectal cancer risk (United States). Cancer Causes Control. 2002; 13:239–248.

58. Kato I, Dnistrian AM, Schwartz M, Toniolo P, Koenig K, Shore RE, Akhmedkhanov A, Zeleniuch-Jacquotte A, Riboli E. Serum folate, homocysteine and colorectal cancer risk in women: a nested case-control study. Br J Cancer. 1999; 79:1917–22. https://doi.org/10.1038/sj.bjc.6690305.

59. Ma, J, Stampfer MJ, Giovannucci E, Artigas C, Hunter DJ, Fuchs C, Willett WC, Selhub J, Hennekens CH, Rozen R. Methylenetetrahydrofolate reductase polymorphism, dietary interactions, and risk of colorectal cancer. Cancer Res. 1997; 57:1098–102.

60. Chen J, Giovannucci E, Kelsey K, Rimm EB, Stampfer MJ, Colditz GA, Spiegelman D, Willett WC, Hunter DJ. Methylenetetrahydrofolate reductase polymorphism and the risk of colorectal cancer. Cancer Res. 1996; 56:4862–4864.

61. Gallegos-Arreola MP, García-Ortiz JE, Figuera LE, Puebla-Pérez AM, Morgan-Villela G, Zúñiga-González GM. Association of the 677C ≥ T polymorphism in the MTHFR gene with colorectal cancer in Mexican patients. Cancer Genomics Proteomics. 2009; 6:183–188.

62. Morita M, Yin G, Yoshimitsu S, Ohnaka K, Toyomura K, Kono S, Ueki T, Tanaka M, Kakeji Y, Maehara Y, Okamura T, Ikejiri K, Futami K, et al. Folate-related nutrients, genetic polymorphisms, and colorectal cancer risk: the fukuoka colorectal cancer study. Asian Pac J Cancer Prev. 2013; 14:6249–6256.

63. Otani T, Iwasaki M, Sasazuki S, Inoue M, Tsugane S. Japan Public Health Center-based Prospective Study Group. Plasma folate and risk of colorectal cancer in a nested case-control study: the Japan Public Health Center-based prospective study. Cancer Causes Control. 2008; 19:67–74. https://doi.org/10.1007/s10552-007-9071-z.

64. Matsuo K, Ito H, Wakai K, Hirose K, Saito T, Suzuki T, Kato T, Hirai T, Kanemitsu Y, Hamajima H, Tajima K. One-carbon metabolism related gene polymorphisms interact with alcohol drinking to influence the risk of colorectal cancer in Japan. Carcinogenesis. 2005; 26:2164–2171. https://doi.org/10.1093/carcin/bgi196.

65. Otani T, Iwasaki M, Hanaoka T, Kobayashi M, Ishihara J, Natsukawa S, Shaura K, Koizumi Y, Kasuga Y, Yoshimura K, Yoshida T, Tsugane S. Folate, vitamin B6, vitamin B12, and vitamin B2 intake, genetic polymorphisms of related enzymes, and risk of colorectal cancer in a hospital-based case-control study in Japan. Nutr Cancer. 2005; 53:42–50. https://doi.org/10.1207/s15327914nc5301_5.

66. Matsuo K, Hamajima N, Hirai T, Kato T, Inoue M, Takezaki T, Tajima K. Methionine Synthase Reductase Gene A66G Polymorphism is Associated with Risk of Colorectal Cancer. Asian Pac J Cancer Prev. 2002; 3:353–359.

67. Kim JW, Jeon YJ, Jang MJ, Kim JO, Chong SY, Ko KH, Hwang SG, Oh D, Oh J, Kim NK. Association between folate metabolism-related polymorphisms and colorectal cancer risk. Mol Clin Oncol. 2015; 3:639–648. https://doi.org/10.3892/mco.2015.520.

68. Kim J, Cho YA, Kim DH, Lee BH, Hwang DY, Jeong J, Lee HJ, Matsuo K, Tajima K, Ahn YO. Dietary intake of folate and alcohol, MTHFR C677T polymorphism, and colorectal cancer risk in Korea. Am J Clin Nutr. 2012; 95:405–412. https://doi.org/10.3945/ajcn.111.020255.

69. Cui LH, Shin MH, Kweon SS, Kim HN, Song HR, Piao JM, Choi JS, Shim HJ, Hwang JE, Kim HR, Park YK, Kim SH. Methylenetetrahydrofolate reductase C677T polymorphism in patients with gastric and colorectal cancer in a Korean population. BMC Cancer. 2010; 10:236. https://doi.org/10.1186/1471-2407-10-236.

70. Takata Y, Shrubsole MJ, Li H, Cai Q, Gao J, Wagner C, Wu J, Zheng W, Xiang YB, Shu XO. Plasma folate concentrations and colorectal cancer risk: a case-control study nested within the Shanghai Men's Health Study. Int J Cancer. 2014; 135:2191–2198. https://doi.org/10.1002/ijc.28871.

71. Yin G, Ming H, Zheng X, Xuan Y, Liang J, Jin X. Methylenetetrahydrofolate reductase C677T gene polymorphism and colorectal cancer risk: A case-control study. Oncol Lett. 2012; 4:365–369. https://doi.org/10.3892/ol.2012.740.

72. Li H, Xu WL, Shen HL, Chen QY, Hui LL, Long LL, Zhu XL. Methylenetetrahydrofolate reductase genotypes and haplotypes associated with susceptibility to colorectal cancer in an eastern Chinese Han population. Genet Mol Res. 2011; 10:3738–3746. https://doi.org/10.4238/2011.December.14.8.

73. Jiang QT, Chen K, Ma XY, Miao XP, Yao KY, Yu WP, Li LY, Zhu YM, Zhou HG. A case-control study on the polymorphisms of methylenetetrahydrofolate reductases, drinking interaction and susceptibility in colorectal cancer. [Article in Chinese]. Zhonghua Liu Xing Bing Xue Za Zhi. 2005; 25:612–616.

74. Chiang FF, Wang HM, Lan YC, Yang MH, Huang SC, Huang YC. High homocysteine is associated with increased risk of colorectal cancer independently of oxidative stress and antioxidant capacities. Clin Nutr. 2014; 33:1054–1060. https://doi.org/10.1016/j.clnu.2013.11.007.

75. Chang SC, Lin PC, Lin JK, Yang SH, Wang HS, Li AF. Role of MTHFR polymorphisms and folate levels in different phenotypes of sporadic colorectal cancers. Int J Colorectal Dis. 2007; 22:483–489.

76. Promthet SS, Pientong C, Ekalaksananan T, Wiangnon S, Poomphakwaen K, Songserm N, Chopjitt P, Moore MA, Tokudome S. Risk factors for colon cancer in Northeastern Thailand: interaction of MTHFR codon 677 and 1298 genotypes with environmental factors. J Epidemiol. 2010; 20:329–338.

77. Sameer AS, Shah ZA, Nissar S, Mudassar S, Siddiqi MA. Risk of colorectal cancer associated with the methylenetetrahydrofolate reductase (MTHFR) C677T polymorphism in the Kashmiri population. Genet Mol Res. 2011; 10:1200–1210. https://doi.org/10.4238/vol10-2gmr1067.

78. Wang J, Gajalakshmi V, Jiang J, Kuriki K, Suzuki S, Nagaya T, Nakamura S, Akasaka S, Ishikawa H, Tokudome S. Associations between 5,10-methylenetetrahydrofolate reductase codon 677 and 1298 genetic polymorphisms and environmental factors with reference to susceptibility to colorectal cancer: a case-control study in an Indian population. Int J Cancer. 2006; 118:991–997. https://doi.org/10.1002/ijc.21438.

79. Tayyem RF, Bawadi HA, Shehadah IN, Abu-Mweis SS, Agraib LM, Bani-Hani KE, Al-Jaberi T, Al-Nusairr M, Heath DD. Macro- and micronutrients consumption and the risk for colorectal cancer among Jordanians. Nutrients. 2015; 7:1769–1786. https://doi.org/10.3390/nu7031769.

80. Arafa MA, Waly MI, Jriesat S, Khafajei AA, Sallam S. Dietary and lifestyle characteristics of colorectal cancer in Jordan: a case-control study. Asian Pac J Cancer Prev. 2011; 12:1931–1936.

81. Naghibalhossaini F, Mokarram P, Khalili I, Vasei M, Hosseini SV, Ashktorab H, Rasti M, Abdollahi K. MTHFR C677T and A1298C variant genotypes and the risk of microsatellite instability among Iranian colorectal cancer patients. Cancer Genet Cytogenet. 2010; 197:142–151. https://doi.org/10.1016/j.cancergencyto.2009.11.014.

82. Haghighi MM, Radpour R, Mohmoudi T, Mohebbi SR, Vahedi M, Zali RM. Association between MTHFR polymorphism (C677T) with nonfamilial colorectal cancer. Oncology Research. 2009; 18:57–63.

83. Williams EA, Welfare M, Spiers A, Hill MH, Bal W, Gibney ER, Duckworth Y, Powers HJ, Mathers JC. Systemic folate status, rectal mucosal folate concentration and dietary intake in patients at differential risk of bowel cancer (The FAB2 Study). Eur J Nutr. 2013; 52:1801–1810. https://doi.org/10.1007/s00394-012-0483-5.

84. Lightfoot TJ, Barrett JH, Bishop T, Northwood EL, Smith G, Wilkie MJ, Steele RJ, Carey FA, Key TJ, Wolf R, Forman D. Methylene tetrahydrofolate reductase genotype modifies the chemopreventive effect of folate in colorectal adenoma, but not colorectal cancer. Cancer Epidemiol Biomarkers Prev. 2008; 17:2421–2430. https://doi.org/10.1158/1055-9965.EPI-08-0058.

85. Al-Ghnaniem R, Peters J, Foresti R, Heaton N, Pufulete M. Methylation of estrogen receptor alpha and mutL homolog 1 in normal colonic mucosa: association with folate and vitamin B-12 status in subjects with and without colorectal neoplasia. Am J Clin Nutr. 2007; 86:1064–1072.

86. Pufulete M, Al-Ghnaniem R, Leather AJ, Appleby P, Gout S, Terry C, Emery PW, Sanders TA. Folate status, genomic DNA hypomethylation, and risk of colorectal adenoma and cancer: a case control study. Gastroenterology. 2003; 124:1240–1248.

87. Wei EK, Giovannucci E, Selhub J, Fuchs CS, Hankinson SE, Ma J. Plasma vitamin B6 and the risk of colorectal cancer and adenoma in women. J Natl Cancer Inst. 2005; 97:684–692. https://doi.org/10.1093/jnci/dji116.

88. Lucock M, Yates Z, Martin C, Choi JH, Beckett E, Boyd L, LeGras K, Ng X, Skinner V, Wai R, Kho J, Roach P, Veysey M. Methylation diet and methyl group genetics in risk for adenomatous polyp occurrence. BBA Clin. 2015; 3:107–112. https://doi.org/10.1016/j.bbacli.2014.11.005.

89. van den Donk M, Buijsse B, van den Berg SW, Ocké MC, Harryvan JL, Nagengast FM, Kok FJ, Kampman E. Dietary intake of folate and riboflavin, MTHFR C677T genotype, and colorectal adenoma risk: a Dutch case-control study. Cancer Epidemiol Biomarkers Prev. 2005; 14:1562–1566. https://doi.org/10.1158/1055-9965.EPI-04-0419.

90. Powers HJ, Hill MH, Welfare M, Spiers A, Bal W, Russell J, Duckworth Y, Gibney E, Williams EA, Mathers JC. Responses of biomarkers of folate and riboflavin status to folate and riboflavin supplementation in healthy and colorectal polyp patients (the FAB2 Study). Cancer Epidemiol Biomarkers Prev. 2007; 16:2128–2135. https://doi.org/10.1158/1055-9965.EPI-07-0208.

91. Mitrou PN, Watson MA, Loktionov AS, Cardwell C, Gunter MJ, Atkin WS, Macklin CP, Cecil T, Bishop TD, Primrose J, Bingham SA. MTHFR (C677T and A1298C) polymorphisms and risk of sporadic distal colorectal adenoma in the UK Flexible Sigmoidoscopy Screening Trial (United Kingdom). Cancer Causes Control. 2006; 17:793–801. https://doi.org/10.1007/s10552-006-0016-8.

92. Tantamango YM, Knutsen SF, Beeson L, Fraser G, Sabate J. Association between dietary fiber and incident cases of colon polyps: the adventist health study. Gastrointest Cancer Res. 2011; 4:161–167.

93. Le Marchand L, Wang, H, Selhub J, Vogt TM, Yokochi L, Decker R. Association of plasma vitamin B6 with risk of colorectal adenoma in a multiethnic case-control study. Cancer Causes Control. 2011; 22:929–936. https://doi.org/10.1007/s10552-011-9759-y.

94. Levine AJ, Lee W, Figueiredo JC, Conti DV, Vandenberg DJ, Davis BD, Edlund CK, Henning SM, Heber D, Stern MC, Haile RW. Variation in folate pathway genes and distal colorectal adenoma risk: a sigmoidoscopy-based case–control study. Cancer Causes Control. 2011; 2254:1–552. https://doi.org/10.1007/s10552-011-9726-7.

95. Ashktorab H, Begum R, Akhgar A, Smoot DT, Elbedawi M, Daremipouran M, Zhao A, Momen B, Giardiello FM. Folate status and risk of colorectal polyps in African Americans. Dig Dis Sci. 2007; 52:1462–1470. https://doi.org/10.1007/s10620-006-9236-8.

96. Hazra A, Wu K, Kraft P, Fuchs CS, Giovannucci EL, Hunter DJ. Twenty-four non-synonymous polymorphisms in the one-carbon metabolic pathway and risk of colorectal adenoma in the Nurses' Health Study. Carcinogenesis. 2007; 28:1510–1519.

97. Boyapati SM, Bostick RM, McGlynn KA, Fina MF, Roufail WM, Geisinger KR, Hebert JR, Coker A, Wargovich M. Folate intake, MTHFR C677T polymorphism, alcohol consumption, and risk for sporadic colorectal adenoma (United States). Cancer Causes Control. 2004; 15:493–501. https://doi.org/10.1023/B:CACO.0000036447.45446.2c.

98. Levine AJ, Siegmund KD, Ervin CM, Diep A, Lee ER, Frankl HD, Haile RW. The methylenetetrahydrofolate reductase 677C->T polymorphism and distal colorectal adenoma risk. Cancer Epidemiol Biomarkers Prev. 2000; 9:657–663.

99. Ulrich CM, Kampman E, Bigler J, Schwartz SM, Chen C, Bostick R, Fosdick L, Beresford SA, Yasui Y, Potter, JD. Colorectal adenomas and the C677T MTHFR polymorphism: evidence for gene-environment interaction? Cancer Epidemiol Biomarkers Prev. 1999; 8:659–668.

100. Chen J, Giovannucci E, Hankinson SE, Ma J, Willett WC, Spiegelman D, Kelsey KT, Hunter DJ. A prospective study of methylenetetrahydrofolate reductase and methionine synthase gene polymorphisms, and risk of colorectal adenoma. Carcinogenesis. 1998; 19:2129–2132.

101. Bird CL, Swendseid ME, Witte JS, Shikany JM, Hunt IF, Frankl HD, Lee ER, Longnecker MP, Haile RW. Red cell and plasma folate, folate consumption, and the risk of colorectal adenomatous polyps. Cancer Epidemiol Biomarkers Prev. 1995; 4:709–714.

102. Yamaji T, Iwasaki M, Sasazuki S, Sakamoto H, Yoshida T, Tsugane S. Methionine synthase A2756G polymorphism interacts with alcohol and folate intake to influence the risk of colorectal adenoma. Cancer Epidemiol Biomarkers Prev. 2009; 18:267–274. https://doi.org/10.1158/1055-9965.EPI-08-0702.

103. Lim YJ, Kim JH, Park SK, Son HJ, Kim JJ, Kim YH. Hyperhomocysteinemia is a risk factor for colorectal adenoma in women. J Clin Biochem Nutr. 2012; 51:132–135. https://doi.org/10.3164/jcbn.D-11-00025.

104. Chiang FF, Huang SC, Wang HM, Chen FP, Huang YC. High serum folate might have a potential dual effect on risk of colorectal cancer. Clin Nutr. 2015; 34:986–990. https://doi.org/10.1016/j.clnu.2014.10.011.

105. Chen FP, Lin CC, Chen TH, Tsai MC, Huang YC. Higher plasma homocysteine is associated with increased risk of developing colorectal polyps. Nutr Cancer. 2013; 65:195–201. https://doi.org/10.1080/01635581.2013.756532.

106. Gao QY, Chen HM, Chen YX, Wang YC, Wang ZH, Tang JT, Ge ZZ, Chen XY, Sheng JQ, Fang DC, Yu CG, Zheng P, Fang JY. Folic acid prevents the initial occurrence of sporadic colorectal adenoma in Chinese older than 50 years of age: a randomized clinical trial. Cancer Prev Res (Phila). 2013; 6:744–752. https://doi.org/10.1158/1940-6207.CAPR-13-0013.

107. Levine AJ, Grau MV, Mott LA, Ueland PM, Baron JA. Baseline plasma total homocysteine and adenoma recurrence: results from a double blind randomized clinical trial of aspirin and folate supplementation. Cancer Epidemiol Biomarkers Prev. 2010; 19:2541–2548. https://doi.org/10.1158/1055-9965.EPI-10-0536.

Supplementary Table 1A: Summary of included studies per blood tests, dietary and lifestyle parameters on the risks of colorectal cancer and adenomas/polyps. See Supplementary_Table_1A

Supplementary Table 1B: Summary of studies with homocysteine levels by MTHFR 677 genotypes and colorectal cancer. See Supplementary_Table_1B

Supplementary Table 2: Pooled meta-analysis: Homocysteine-related blood test measurements on the risks of colorectal cancer and adenomas/polyps (AP). See Supplementary_Table_2

Supplementary Table 3A: Summary of studies with homocysteine levels by MTHFR 677 genotypes and colorectal cancer. See Supplementary_Table_3A

Supplementary Table 3B: Pooled meta-analysis: Dietary parameters on the risks of colorectal cancer (CRC) and adenomas/polyps (AP). See Supplementary_Table_3B

Supplementary Table 4A: Pooled meta-analysis: Lifestyle factors on the risks of colorectal cancer and adenomas/polyps (effect size). See Supplementary_Table_4A

Supplementary Table 4B: Pooled meta-analysis: Lifestyle on the risks of colorectal cancer and adenomas/polyps. See Supplementary_Table_4B
