# Supplementary material for: Meta-analysis of homocysteine-related factors on the risk of colorectal cancer
Source: Oncotarget. 2018 May 22;9(39):25681–97. doi: 10.18632/oncotarget.25355 (PMC5986656; doi:10.18632/oncotarget.25355)
Supplement: Supplementary file 2 [file oncotarget-09-25681-s002.docx]

Supplementary Table 1A: Summary of included studies per blood tests, dietary and lifestyle parameters on the risks of colorectal cancer (CRC) and adenomas/polyps (AP). (86 papers: 63 CRC, 5 CRC and AP, 18 AP)

| First Author, Year  (Reference Number) | Ethnicity - Country | CS^D^ | Plasma | | | | | | Dietary | | | | | | | | | | | Lifestyle | | Quality  Score^a^ | Design |
| --- | --- | --- | --- | --- | --- | --- | --- | --- | --- | --- | --- | --- | --- | --- | --- | --- | --- | --- | --- | --- | --- | --- | --- |
|  |  |  | Hcy | B12 | Met | B9 | B6 | B2 | B12 | Met | B9 | Supp  B9 | B6 | Supp  B6 | B2 | Fiber | Veg | Red Meat | Multi-Vitamin | Alcohol | Smoking |  |  |
| **Colorectal Cancer** |  |  |  |  |  |  |  |  |  |  |  |  |  |  |  |  |  |  |  |  |  |  |  |
| Nitter, 2014 (20) | European – 10 countries^b^ | 4^P^ |  |  | M |  |  |  |  |  |  |  |  |  |  |  |  | M |  |  |  | 22 (8, 7, 7) | Cohort (EPIC) |
| Eussen, 2010a (21) | European – 10 countries^b^ | 4^P^ |  | M |  |  | M | M |  |  |  |  |  |  |  |  |  |  |  | C |  | 22 (8, 7, 7) | Cohort (EPIC) |
| Eussen, 2010b (22) | European – 10 countries | 4^H^ | M^c^ |  |  | M |  |  |  |  |  |  |  |  |  |  |  |  |  |  | C | 23^&^ (8, 8, 7) | Cohort (EPIC) |
| Iacopetta, 2009 (23) | European – Australia | 4^H^ |  |  |  |  |  |  |  |  | C |  |  |  |  |  |  |  |  | C | C | 20 (9, 5, 6) | Case-Control |
| Weinstein, 2008 (24) | European – Finland | 3^+^ |  |  |  |  |  |  |  |  |  |  |  |  |  |  |  |  |  |  |  | 21 (7, 7, 7) | Cohort (ABCPS) |
|  | Colon |  | M | M |  | M | M | M | M | M | M | C | M | C |  |  |  |  |  | M |  |  |  |
|  | Rectum |  | M | M |  | M | M | M | M | M | M | C | M | C |  |  |  |  |  | M |  |  |  |
| Osian, 2007 (25) | European – Romania | 4^H^ |  |  |  |  |  |  |  |  |  |  |  |  |  |  |  |  |  | C | C | 21 (9, 7, 5) | Case-Control |
| Komlosi, 2010 (26) | European – Hungary | 3^M^ |  |  |  |  |  |  |  |  |  |  |  |  |  |  |  |  |  |  |  | 20 (6, 6, 8) | Case-Control |
|  | Colon |  | M |  |  |  |  |  |  |  |  |  |  |  |  |  |  |  |  |  |  |  |  |
|  | Rectum |  | M |  |  |  |  |  |  |  |  |  |  |  |  |  |  |  |  |  |  |  |  |
| Pardini, 2011 (27) | European – Czech Republic | 4^H^ |  |  |  |  |  |  |  |  |  |  |  |  |  |  |  |  |  |  | C | 19 (6, 7, 6) | Case-Control |
| Myte, 2016 (28) | European – Sweden | 4^HP^ |  |  | M |  |  |  |  |  |  |  |  |  |  |  |  |  |  |  |  | 20 (7, 6, 7) | Case-Control |
| Gylling, 2014 (29) | European – Sweden | 4^M^ | M | M |  | M |  |  |  |  |  |  |  |  |  |  |  |  |  |  | C | 21 (7 ,7, 7) | Case-Control |
| Dahlin, 2008 (30) | European – Sweden | 4^HP^ |  | M |  |  |  |  |  |  |  |  |  |  |  |  |  |  |  |  |  | 19 (7, 6, 6) | Cohort (NSHDS) |
| Ulvik, 2004 (31) | European – Norway | 4^HP^ |  |  |  |  |  |  |  |  |  |  |  |  |  |  |  |  |  |  | C | 17 (7, 5, 5) | Cohort (JANUS) |
| Heijmans, 2003 (32) | European – Netherlands | 4^M^ |  |  |  |  |  |  |  |  | C |  |  |  |  |  |  |  |  | C |  | 18 (6, 5, 7) | Case-Control |
| Vossen, 2011 (33) | European – Germany | 4^H^ |  |  |  |  |  |  |  |  |  |  |  |  |  |  |  |  |  |  | C | 21 (9, 6, 6) | Case-Control |
| Ferroni, 2009 (34) | European – Italy | 4^M^ | M | M |  | M |  |  |  |  |  |  |  |  |  |  |  |  |  |  |  | 20 (5, 7, 8) | Case-Control |
| Battistelli, 2006 (35) | European – Italy | 4^HP^ | M^c^ |  |  |  |  |  |  |  |  |  |  |  |  |  |  |  |  |  |  | 17^&^ (6, 5, 6) | Case-Control |
| Theodoratou, 2008 (36) | European – UK | 4^M^ |  |  |  |  |  |  |  |  | MC |  | C |  |  | M |  |  |  | M | C | 17 (7, 4, 6) | Case-Control |
| Sharp, 2007 (37) | European – UK | 4^H^ |  |  |  |  |  |  |  |  |  |  |  |  |  |  |  |  |  | C | C | 18 (7, 4, 7) | Case-Control |
| Guerreiro, 2008 (38) | European – Portugal | 4^H^ |  |  |  |  |  |  |  | M |  |  |  |  |  |  |  |  |  | M |  | 15 (6, 3, 6) | Case-Control |
| Ulrich, 2013 (39) | Caucasian – Germany, US | 4^P^ |  | M |  | M |  |  |  |  |  |  |  |  |  |  |  |  |  |  |  | 23 (9, 6, 8) | Case-Control |
| Levine, 2010 (40) | Caucasian – Australia, Canada, US | 4^M^ |  |  |  |  |  |  |  |  |  |  |  |  |  |  |  |  |  |  |  | 17 (6, 5, 6) | Case-Control |
|  | Population based |  |  |  |  |  |  |  | M |  | M | C | M |  |  |  |  |  | C | C | C |  |  |
|  | Clinic based |  |  |  |  |  |  |  | M |  | M | C | M |  |  |  |  |  | C | C | C |  |  |
| Sun, 2012 (41) | Caucasian - Canada | 4^P^ |  |  |  |  |  |  |  |  |  |  |  |  |  | M |  |  |  | M |  | 17 (8, 3, 6) | Case-Control |
| Navarro, 2016 (42) | Caucasian – US | 4^M^ |  |  |  |  |  |  |  |  | M |  |  |  |  | M |  | M |  | M | C | 15 (5, 3, 7) | Cohort (WHI) |
| Cheng, 2015 (43) | Caucasian – US | 4^M^ | M | M |  | M | M |  |  |  |  |  |  |  |  |  |  |  |  |  | M | 20 (6, 7, 7) | Cohort (WHI) |
| Cho, 2015 (44) | Caucasian – US | 4^M^ |  |  |  |  |  |  |  |  |  |  |  |  |  |  |  |  | C |  | C | 21 (6, 7, 8) | Cohort (NHS,HPFS) |
| Neuhousser, 2015 (45) | Caucasian – US | 4^M^ |  |  |  |  |  |  |  |  | M |  |  |  |  |  |  |  |  |  | M | 23 (9, 7, 7) | Cohort (WHI) |
| Ashmore, 2013 (46) | Caucasian – US | 4^H^ |  |  |  |  |  |  |  | M | M | MC |  |  |  | M |  |  |  | M | C | 19 (6, 6, 7) | Case-Control |
| Miller, 2013 (47) | Caucasian – US | 4^M^ | M |  |  |  |  |  |  |  |  |  |  |  |  |  |  |  |  |  |  | 21 (8, 7, 6) | Cohort (WHI) |
| Zschabitz, 2012 (48) | Caucasian – US | 4^M^ |  |  |  |  |  |  |  |  | C |  |  |  |  |  |  |  |  |  |  | 17 (6, 5, 6) | Cohort (WHI) |
| Curtin, 2011 (49) | Caucasian – US | 2^P^ |  |  |  |  |  |  |  | C |  |  |  |  |  |  |  |  | C |  |  | 19 (8, 4, 7) | Case-Control |
| Le Marchand, 2009 (50) | Caucasian – US | 4^M^ | M | M |  | M |  |  |  |  |  |  |  |  |  | M |  |  | C | M | MC | 20 (7, 6, 7) | Cohort (MEC) |
| Lee, 2009 (51) | Caucasian – US | 4^M^ | M | M |  | M |  |  |  |  |  |  |  |  |  |  |  | C | C | C | C | 19 (5, 7, 7) | Cohort (Physician Health Study) |
| Murtaugh, 2007 (52) | Caucasian – US | 2^P^ |  |  |  |  |  |  | C | C |  |  | C |  | C |  |  |  |  |  |  | 19 (7, 5, 7) | Case-Control |
|  | Male |  |  |  |  |  |  |  | M | M |  |  | M |  | M | M |  |  |  |  |  |  |  |
|  | Female |  |  |  |  |  |  |  | M | M |  |  | M |  | M | M |  |  |  |  |  |  |  |
| Koushik, 2006 (53) | Caucasian – US | 4^M^ |  |  |  |  |  |  |  |  |  |  |  |  |  |  |  |  |  |  |  | 19 (7, 5, 7) | Cohort (NHS,HPFS) |
|  | Male |  |  |  |  |  |  |  |  |  |  |  |  |  |  |  |  | C |  |  |  |  |  |
|  | Female |  |  |  |  |  |  |  |  |  |  |  |  |  |  |  |  | C |  |  |  |  |  |
| Le Marchand, 2005 (54) | Caucasian – US | 4^M^ |  |  |  |  |  |  |  |  | MC |  |  |  |  |  |  | M |  | MC | M | 20 (8, 6, 6) | Cohort (MEC) |
| Curtin, 2004 (55) | Caucasian – US | 1^H^ |  |  |  |  |  |  |  |  |  |  |  |  |  |  |  |  |  |  |  | 19 (8, 5, 6) | Case-Control |
|  | Male |  |  |  |  |  |  |  | MC |  | MC | M |  |  |  |  |  |  |  | MC |  |  |  |
|  | Female |  |  |  |  |  |  |  | MC |  | MC | M |  |  |  |  |  |  |  | MC |  |  |  |
| Keku, 2002 (56) | Caucasian – US | 1^H^ |  |  |  |  |  |  |  |  |  |  |  |  |  |  |  |  |  |  | C | 18 (6, 5, 7) | Case-Control |
|  | African American – US |  |  |  |  |  |  |  |  |  |  |  |  |  |  |  |  |  |  |  | C |  |  |
| Le Marchand, 2002 (57) | Caucasian – US | 4^M^ |  |  |  |  |  |  |  | M |  |  |  | M |  |  | M |  |  | M | M | 20 (7, 6, 7) | Case-Control |
| Kato, 1999 (58) | Caucasian – US | 4^P^ |  |  |  |  |  |  |  |  |  |  |  |  |  |  |  |  | C |  |  | 20 (7, 7, 6) | Cohort (NYUWHS) |
| Ma, 1997 (59) | Caucasian – US | 4^M^ |  |  |  |  |  |  |  |  |  |  |  |  |  |  |  |  | C | C | C | 23 (8, 7, 8) | Cohort (PHS) |
| Chen, 1996 (60) | Caucasian – US | 4^M^ |  |  |  |  |  |  |  | C | C |  |  |  |  |  |  |  |  | C |  | 16 (5, 5, 6) | Cohort |
| Gallegos-Arreola, 2009 (61) | Hispanic - Mexico | 4^P^ |  |  |  |  |  |  |  |  |  |  |  |  |  |  |  |  |  |  | C | 16 (6, 5, 5) | Case-Control |
| Morita, 2014 (62) | East Asian – Japan | 4^H^ |  |  |  |  |  |  |  |  |  |  |  |  |  |  |  |  |  | C | C | 18 (7, 5, 6) | Case-Control |
| Otani, 2008 (63) | East Asian – Japan | 4^H^ |  |  |  |  |  |  |  |  |  |  |  |  |  |  |  |  |  |  |  | 22 (7, 8, 7) | Cohort (HPFS) |
|  | Male |  |  |  |  |  |  |  |  |  |  |  |  |  |  |  |  |  | C |  |  |  |  |
|  | Female |  |  |  |  |  |  |  |  |  |  |  |  |  |  |  |  |  | C |  |  |  |  |
| Matsuo, 2005 (64) | East Asian – Japan | 4^H^ |  |  |  |  |  |  |  |  | MC |  |  |  |  |  |  |  |  | C | C | 18 (8, 4, 6) | Case-Control |
| Otani, 2005 (65) | East Asian – Japan | 4^HP^ |  |  |  |  |  |  | MC |  | MC |  | MC |  | MC | M |  | M |  | M | C | 20 (7, 6, 7) | Case-Control |
| Matsuo, 2002 (66) | East Asian – Japan | 4^H^ |  |  |  |  |  |  |  |  |  |  |  |  |  |  |  |  |  |  |  | 17 (7, 4, 6) | Case-Control |
|  | Male |  |  |  |  |  |  |  |  |  |  |  |  |  |  |  |  |  |  |  | C |  |  |
|  | Female |  |  |  |  |  |  |  |  |  |  |  |  |  |  |  |  |  |  |  | C |  |  |
| Kim, 2015 (67) | East Asian – Korea | 4^P^ | M |  |  | M |  |  |  |  |  |  |  |  |  |  |  |  |  |  |  | 18 (7, 5, 6) | Case-Control |
| Kim, 2012 (68) | East Asian – Korea | 4^P^ |  |  |  |  |  |  |  |  | MC |  |  |  |  |  |  |  | C | C | C | 19 (7, 6, 6) | Case-Control |
| Cui, 2010 (69) | East Asian – Korea | 4^P^ |  |  |  |  |  |  |  |  |  |  |  |  |  |  |  |  |  | C | C | 15 (6, 3, 6) | Case-Control |
| Takata, 2014 (70) | East Asian – China | 4^M^ |  |  |  | M |  |  | M | M | M |  | M |  | M |  |  |  | C |  | C | 22 (7, 8, 7) | Cohort (SMHS) |
| Yin, 2012 (71) | East Asian – China | 4^H^ | M^c^ |  |  |  |  |  |  |  |  |  |  |  |  |  |  |  |  |  |  | 19^&^ (7, 6, 6) | Case-Control |
| Li, 2011 (72) | East Asian – China | 4^HP^ |  |  |  |  |  |  |  |  |  |  |  |  |  |  |  |  |  | C | C | 15 (6, 3, 6) | Case-Control |
| Jiang, 2005 (73) | East Asian – China | 4^M^ |  |  |  |  |  |  |  | M | M |  |  |  |  |  |  |  |  | C | C | 16 (6, 4, 6) | Cohort |
| Chiang, 2014 (74) | East Asian – Taiwan | 4^M^ | M |  |  | M |  |  |  |  |  |  |  |  |  |  |  |  |  | C | C | 17 (6, 4, 7) | Case-Control |
| Chang, 2007 (75) | East Asian – Taiwan | 4^P^ |  | M |  | M |  |  |  |  |  |  |  |  |  |  |  |  |  |  |  | 20 (8, 6, 6) | Case-Control |
| Promthet, 2010 (76) | South Asian – Thailand | 1^H^ |  |  |  |  |  |  |  |  |  |  |  |  |  |  |  | C |  | C | C | 18 (7, 6, 5) | Case-Control |
| Sameer, 2011 (77) | South Asian – India | 4^P^ |  |  |  |  |  |  |  |  |  |  |  |  |  |  |  |  |  |  | C | 17 (7, 4, 6) | Case-Control |
| Wang, 2006 (78) | South Asian – India | 4^M^ |  |  |  |  |  |  |  |  |  |  |  |  |  |  |  |  |  | C | C | 17 (6, 5, 6) | Case-Control |
| Tayyem, 2015 (79) | Middle Eastern – Jordan | 4^P^ |  |  |  |  |  |  | M |  | M |  |  |  |  | M |  |  |  |  |  | 19 (6, 6, 7) | Case-Control |
| Arafa, 2011 (80) | Middle Eastern – Jordan | 4^H^ |  |  |  |  |  |  |  |  |  |  |  |  |  |  |  |  |  |  | C | 18 (6, 6, 6) | Case-Control |
| Naghibalhossaini, 2010 (81) | Middle Eastern – Iran | 1^H^ |  |  |  |  |  |  |  |  |  |  |  |  |  |  |  |  |  |  | C | 15 (6, 3, 6) | Case-Control |
| Haghighi, 2009 (82) | Middle Eastern – Iran | 4^P^ |  |  |  | M |  |  |  |  | C |  |  |  |  |  |  |  |  |  |  | 22 (7, 8, 7) | Case-Control |
| **Colorectal Cancer & Adenomas/Polyps** | |  |  |  |  |  |  |  |  |  |  |  |  |  |  |  |  |  |  |  |  |  |  |
| Williams, 2013 (83) | European – UK | 2^P^ |  |  |  |  |  |  |  |  |  |  |  |  |  |  |  |  |  |  |  | 17 (5, 5, 7) | Case-Control |
|  | CRC |  | M | M |  | M |  |  |  |  | M |  |  |  | M |  |  |  |  |  | C |  |  |
|  | AP |  | M | M |  | M |  |  |  |  | M |  |  |  | M |  |  |  |  |  | C |  |  |
| Lightfoot, 2008 (84) | European – UK | 4^P^ |  |  |  |  |  |  |  |  |  |  |  |  |  |  |  |  |  |  |  | 18 (6, 6, 6) | Case-Control |
|  | CRC |  |  |  |  |  |  |  |  |  |  |  |  |  |  |  |  |  |  | C |  |  |  |
|  | Adenoma |  |  |  |  |  |  |  |  |  |  |  |  |  |  |  |  |  |  | C |  |  |  |
| Al-Ghnaniem, 2007 (85) | European – UK | 4^H^ |  |  |  |  |  |  |  |  | M |  |  |  |  |  |  |  |  |  |  | 19 (5, 7, 7) | Case-Control |
|  | CRC |  |  |  |  |  |  |  |  |  | M |  |  |  |  |  |  |  |  | M |  |  |  |
|  | AP |  |  |  |  |  |  |  |  |  | M |  |  |  |  |  |  |  |  |  |  |  |  |
|  | Adenoma |  |  |  |  |  |  |  |  |  |  |  |  |  |  |  |  |  |  | M |  |  |  |
|  | Polyp |  |  |  |  |  |  |  |  |  |  |  |  |  |  |  |  |  |  | M |  |  |  |
| Pufulete, 2003 (86) | European – UK | 4^H^ |  |  |  |  |  |  |  |  |  |  |  |  |  |  |  |  |  |  |  | 20 (5, 8, 7) | Case-Control |
|  | CRC |  | M | M |  | M |  |  |  |  |  | C |  |  |  |  |  |  |  | C | C |  |  |
|  | AP |  | M | M |  | M |  |  |  |  |  | C |  |  |  |  |  |  |  | C | C |  |  |
| Wei, 2005 (87) | Caucasian – US | 4^M^ |  |  |  |  |  |  |  |  |  |  |  |  |  |  |  |  |  |  |  | 22 (6, 8, 8) | Cohort (NHS) |
|  | CRC |  | M | M |  | M | M |  | M | M | M |  | M |  |  |  |  |  | C | M | M |  |  |
|  | AP |  | M | M |  | M | M |  | M | M | M |  | M |  |  |  |  |  | C | M | M |  |  |
| **Adenomas/Polyps** |  |  |  |  |  |  |  |  |  |  |  |  |  |  |  |  |  |  |  |  |  |  |  |
| Lucock, 2015 (88) | European - Australia | 4^P^ | M |  |  |  |  |  | M | M | M |  | M |  |  |  |  |  |  |  |  | 16 (5, 6, 5) | Case-Control |
| Van den Donk, 2005 (89) | European – Netherlands | 4^H^ |  |  |  |  |  |  | M |  | M |  | M |  | M | M | M |  | C | M | C | 17 (6, 5, 6) | Case-Control |
| Powers, 2007 (90) | European – UK | 4^HP^ | M | M |  | M |  | M |  |  |  |  |  |  |  |  |  |  |  |  |  | 17 (5, 5, 7) | Cohort & RCT |
| Mitrou, 2006 (91) | European – UK | 5^H^ |  |  |  |  |  |  |  |  |  |  |  |  |  |  |  |  |  |  | C | 20 (9, 5, 6) | Case-Control |
| Tantamango, 2011 (92) | Caucasian – US/Canada | 4^M^ |  |  |  |  |  |  |  |  |  |  |  |  |  | M | M |  |  | C |  | 14 (5, 3, 6) | Cohort (AHS-1 & 2) |
| Le Marchand, 2011 (93) | Caucasian – US | 4^H^ |  |  |  |  |  |  |  |  |  |  |  |  |  |  |  |  | C | M | MC | 19 (6, 6, 7) | Case-Control |
| Levine, 2011 (94) | Caucasian – US | 4^H^ | M |  |  |  |  |  | M |  | M |  |  |  |  | M | M | M | C | M | MC | 21 (7, 7, 7) | Case-Control |
| Ashktorab, 2007 (95) | Caucasian – US | 4^H^ | M | M |  | M |  |  |  |  |  |  |  |  |  |  |  |  |  |  |  | 19 (6, 7, 6) | Case-Control |
| Hazra, 2007 (96) | Caucasian – US | 4^+^ |  |  |  |  |  |  |  | M | MC |  |  |  |  |  |  | C | C | MC | C | 21 (7, 7, 7) | Cohort (NHS) |
| Boyapati, 2004 (97) | Caucasian – US | 4^P^ |  |  |  |  |  |  |  | MC |  |  |  |  |  | M |  |  |  | C | C | 16 (6, 4, 6) | Case-Control |
| Levine, 2000 (98) | Caucasian – US | 5^H^ |  |  |  | M |  |  |  |  |  |  |  |  |  |  |  |  |  | C |  | 21 (7, 8, 6) | Case-Control |
| Ulrich, 1999 (99) | Caucasian – US | 4^H^ |  |  |  |  |  |  | M | M | M |  | M |  |  | M |  |  |  | MC |  | 16 (5, 6, 5) | Case-Control |
| Chen, 1998 (100) | Caucasian – US | 4^M^ |  |  |  |  |  |  |  |  | C |  |  |  |  |  |  |  |  |  |  | 16 (6, 5, 5) | Cohort (NHS) |
| Bird, 1995 (101) | Caucasian – US | 4^H^ |  |  |  |  |  |  |  |  |  |  |  |  |  |  |  |  |  |  |  | 20 (6, 7, 7) | Case-Control |
|  | Male |  |  |  |  |  |  |  |  | M |  | C |  |  |  |  |  |  |  |  |  |  |  |
|  | Female |  |  |  |  |  |  |  |  | M |  | C |  |  |  |  |  |  |  |  |  |  |  |
| Yamaji, 2009 (102) | East Asian – Japan | 4^P^ |  |  |  |  |  |  |  |  |  |  |  |  |  |  |  |  |  | C | C | 16 (5, 5, 6) | Case-Control |
| Lim, 2012 (103) | East Asian – South Korea | 4^H^ | M |  |  |  |  |  |  |  |  |  |  |  |  |  |  |  |  | C | C | 17 (6, 6, 5) | Case-Control |
| Chiang, 2015 (104) | East Asian - Taiwan | 4^H^ | M |  |  | M |  |  |  |  |  |  |  |  |  |  |  |  |  |  | C | 15 (5, 4, 6) | Case-Control |
| Chen, 2013 (105) | East Asian - Taiwan | 4^H^ | M | M |  | M |  |  |  |  |  |  |  |  |  |  |  |  |  |  | C | 19 (6, 6, 7) | Case-Control |
| **Additional RCT** |  |  |  |  |  |  |  |  |  |  |  |  |  |  |  |  |  |  |  |  |  |  |  |
| Gao, 2013 (106) |  | 4^P^ |  |  |  | C |  |  |  |  |  |  |  |  |  |  |  |  |  |  |  | 17 (7, 5, 5) | RCT^^1^ |
| Levine, 2010 (107) |  | 4^H^ | R |  |  |  |  |  |  |  |  |  |  |  |  |  |  |  |  |  |  | 20 (7, 8, 5) | RCT^^2^ |

*Notes:* Cancer Site (CS): 1 = colon (C), 2 = rectum (R), 3 = CR each, 4 = CR mixed, 5 = distal; ^D^ Diagnosis methods: ^H^ histologically, ^P^ pathologically, ^M^ medical record, ^+^ all three methods. M: Mean and standard deviation, C: Counts, R: Range. Hcy=Homocysteine; Met=Methionine; B12, B9, B6, B2=Vitamins; Veg= Vegetables; UK= United Kingdom; US=United States of America. EPIC= European Prospective Investigation into Cancer and Nutrition; ABCPS= Alpha-Tocopherol, Beta-Carotene Cancer Prevention Study; NSHDS= Northern Sweden Health and Disease Study; JANUS=Janus Serum Bank Cohort; WHI=Women’s Health Initiative Observation Study; NHS=Nurse’s Health Study; HPFS=Health Professionals Follow-up Study; MEC=Multiethnic Cohort Study; NYUWHS=New York University Women’s Health Study; PHS=Physician’s Health Study; SMHS= Shanghai Men’s Health Study; AHS 1 & 2=Adventist Health Study 1 & 2; ^a^ Quality Score Ranges = 0-28 (External validity=0-9, Internal Validity=0-9, Report Quality=0-10); ^&^ Study includes DNA analysis for MTHFR 677 genotype. ^b^ 10 European Countries: Denmark, France, Greece, Germany, Italy, Netherlands, Norway, Spain, Sweden, United Kingdom. RCT=Randomized controlled trial. ^^1^: 1 mg folic acid 3 years, ^^2^: 1 mg folic acid 3 months.
