# Supplementary material for: Meta-analysis of homocysteine-related factors on the risk of colorectal cancer
Source: Oncotarget. 2018 May 22;9(39):25681–97. doi: 10.18632/oncotarget.25355 (PMC5986656; doi:10.18632/oncotarget.25355)
Supplement: Supplementary file 3 [file oncotarget-09-25681-s003.docx]

Supplementary Table 1B: Summary of studies with homocysteine levels (mmol/L) by *MTHFR* 677 genotypes and colorectal cancer

(2 papers).

| First Author, Year  (Reference Number) | Ethnicity  - Country | *MTHFR* 677 | | | | | | Quality  Score^a^ |
| --- | --- | --- | --- | --- | --- | --- | --- | --- |
|  |  | CC | | CT | | TT | |  |
|  |  | Case  n (%)  Mean + SD | Control  n (%)  Mean + SD | Case  n (%)  Mean + SD | Control  n (%)  Mean + SD | Case  n (%)  Mean + SD | Control  n (%)  Mean + SD |  |
| Battistelli, 2006 (40) | European – Italy | 32 (21)  11.82 ± 3.09 | 30 (18)  9.01 ± 2.76 | 40 (19)  12.45 ± 4.46 | 51 (22)  9.71 ± 2.34 | 21 (21)  16.88 ± 9.25 | 19 (26)  17.56 ± 10.81 | 17  (6, 5^PCR^, 6) |
| Yin, 2012 (75) | East Asian – China | 124 (79)  11.67 ± 2.03 | 139 (82)  9.54 ± 1.72 | 167 (81)  12.94 ± 2.68 | 178 (78)  9.98 ± 2.33 | 79 (79)  15.36 ± 3.44 | 53 (74)  13.79 ± 3.85 | 19  (7, 6 ^PCR^, 6) |
| Excluded for missing data | |  |  |  |  |  |  |  |
| Ma, 1999 (16) | Caucasian – US | 79 (34)  12 + -- | 135 (9)  12.2 + -- | 78 (27)  12.3 + -- | 111 (9)  11.8 + -- | 13 (11)  17.6 + -- | 43 (12)  13.1 + -- | 22  (9, 7 ^PCR^, 6) |
| Eussen, 2010b (27) | European – 10 countries^b^ | -- | 1131 (79)  10.1 + -- | -- | 933 (73)  10.2 + -- | -- | 254 (69)  11.9 + -- | 23  (8, 8 ^MAL^, 7) |

*Notes:* mmol/L: micromole per liter; *MTHFR:* *methyltetrahydrofolate reductase*; US=United States of America

^a^ Quality Score Ranges = 0-28 (External validity=0-9, Internal Validity=0-9 (*MTHFR* genotyping method: ^PCR^ polymerase chain reaction, ^MAL^ matrix-assisted laser desorption/ionization), Report Quality=0-10).
^b^10 European Countries: Denmark, France, Greece, Germany, Italy, Netherlands, Norway, Spain, Sweden, United Kingdom

--: No data
