# Supplementary material for: Meta-analysis of homocysteine-related factors on the risk of colorectal cancer
Source: Oncotarget. 2018 May 22;9(39):25681–97. doi: 10.18632/oncotarget.25355 (PMC5986656; doi:10.18632/oncotarget.25355)
Supplement: Supplementary file 4 [file oncotarget-09-25681-s004.docx]

Supplementary Table 2. Pooled meta-analysis: Homocysteine-related blood test measurements on the risks of colorectal cancer (CRC) and adenomas/polyps (AP) (37 studies).

| Plasma measurements  (Number of studies) | Case  N=8,401  Mean + SD  (Range) | Control N=11,009  Mean + SD  (Range) | Test of Heterogeneity | | | Test of Association | |
| --- | --- | --- | --- | --- | --- | --- | --- |
|  |  |  | Q | *p* | *I^2^* (%) | Pooled Effect Size  (95% Cl) | *p* |
| Homocysteine (28) mmol/L | 8,401  12.44 + 2.86  (7.7 – 20.8) | 11,009  11.38 + 2.33  (7 – 17.9) | 826.26 | <0.0001 | 96.7 | 0.62 (0.48 – 0.79) | <0.0001 |
| Case-Control (19) | 3,844  13.24 + 2.97  (7.7 – 20.8) | 5,042  11.81 + 2.52  (7 – 17.9) | 692.53 | <0.0001 | 97.4 | 0.92 (0.64 – 1.21) | <0.0001 |
| CRC (10) | 2,458  13.65 + 3.99  (7.7 – 20.8) | 2,981  11.95 + 3.4  (7 – 17.9) | 244.93 | <0.0001 | 96.3 | 0.71 (0.41 – 1.02) | <0.0001 |
| European (7) | 1,478  13.93 + 4.62  (7 – 20.8) | 1,915  12.84 + 3.99  (7 – 17.9) | 138.27 | <0.0001 | 95.7 | 0.76 (0.39 – 1.14) | <0.0001 |
| East Asian (3) | 980  13.01 + 2.6  (10.41 – 15.6) | 1,066  10.7 + 0.87  (10.06 – 11.69) | 96.91 | <0.0001 | 97.9 | 0.62 (-0.03 – 1.26) | 0.0617 |
| AP (9) | 1,486  12.78 + 1.22  (10.48 – 14.31) | 2,061  11.65 + 1.12  (9.8 – 13.16) | 444.02 | <.0001 | 98.2 | 1.13 (0.57 – 1.77) | 0.0001 |
| European (4) | 268  13.12 + 0.5  (12.4 – 13.49) | 435  12.25 + 0.45  (11.7 – 12.69) | 139.32 | <.0001 | 97.8 | 2.27 (0.87 – 3.68) | 0.0015 |
| Caucasian (2) | 678  10.84 + 0.51  (10.48 – 11.2) | 731  10.63 + 0.39  (10.35 – 10.9) | 0.03 | 0.8952 | 0 | 0.032 (-0.07 – 0.14) | 0.549 |
| East Asian (3) | 540  13.63 + 0.59  (13.28 – 14.31) | 895  11.53 + 1.68  (9.8 – 13.16) | 31.76 | <0.0001 | 93.7 | 0.47 (-0.10 – 1.04) | 0.1061 |
| Cohort (9) | 4,457  10.74 + 1.7  (8.81 – 13.67) | 5,967  10.47 + 1.64  (8.39 – 13.33) | 34.23 | <0.0001 | 76.6 | 0.09 (0.0 – 0.18) | 0.0429 |
| CRC (8) | 4,047  10.86 + 1.79  (8.81 – 13.67) | 5,557  10.52 + 1.74  (8.39 – 13.33) | 29.33 | 0.0001 | 76.1 | 0.11 (0.02 – 0.21) | 0.0169 |
| European (3) | 1,645  12.4 + 1.91  (10.2 – 13.67) | 2,603  12.18 + 1.64)  (10.3 – 13.33) | 2.96 | 0.228 | 32.4 | -0.02 (-0.09 – 0.04) | 0.4324 |
| Caucasian (5) | 2,402  9.94 + 0.93  (8.81 – 11) | 2,954  9.54 + 0.82  (8.39 – 10.33) | 4.37 | 0.3578 | 8.5 | 0.17 (0.12 – 0.23) | <0.0001 |
| AP (1) | 410  9.8 + 2.59 | 410  10 + 2.74 | -- | -- | -- | -- | -- |
|  |  |  |  |  |  |  |  |
| Vitamin B12 (20)  pmol/L | 4,764  390.2 + 103.6 (257 – 638) | 6,966  401.66 + 82.58 (288 – 531.3) | 528.38 | <0.0001 | 96.4 | -0.55 (-0.77 – -0.33) | <0.0001 |
| Case-Control (11) | 1,059  383.78 + 118.27  (257 – 638) | 1,550  394.55 + 79.99  (302 – 531.3) | 476.57 | <0.0001 | 97.9 | -1.08 (-1.72 – -0.45) | 0.0009 |
| CRC (6) | 774  391.58 + 137.31  (257 – 638) | 1,164  397.41 + 83.47  (302 – 515) | 224.03 | <0.0001 | 97.8 | -0.99 (-1.74 - -0.25) | 0.0086 |
| European (5) | 579  383.9 + 152.09  (257 – 638) | 969  387.5 + 89.61  302 – 515 | 220.82 | <0.0001 | 98.2 | -1.21 (-2.26 - -0.16) | 0.0244 |
| East Asian (1) | 195  430 + 174 | 195  445 + 250 | -- | -- | -- | -- | -- |
| AP (5) | 285  374.42 + 105.8  (306 – 561.3) | 386  391.12 + 85.21  (311.3 – 531.3) | 223.47 | <0.0001 | 98.2 | -1.18 (-2.54 – 0.18) | 0.0881 |
| European (3) | 214  322.96 + 20.61  (306 – 345.9) | 255  350.43 + 45.26  (311.3 – 400) | 187.05 | <0.0001 | 98.9 | -1.96 (-4.27 – 0.35) | 0.096 |
| Caucasian (1) | 23  561.3 + 311.7 | 35  531.3 + 264.6 | -- | -- | -- | -- | -- |
| East Asian (1) | 48  341.91 + 178.89 | 96  373 + 205.4 | -- | -- | -- | -- | -- |
| Cohort (9) | 3705  398.04 + 88.78  (286.67 – 517) | 5416  410.33 + 89.69  (288 – 529) | 11.25 | 0.1879 | 28.9 | -0.05 (-0.09 – 0) | 0.0242 |
| CRC (8) | 3299  394.63 + 94.27  (286.67 – 517) | 5010  406.625 + 95.15  (288 – 529) | 10.7 | 0.1523 | 34.6 | -0.04 (-0.09 – 0) | 0.0551 |
| European (4) | 1866  313.17 + 30.23  (286.67 – 345) | 3030  326.5 + 41.11  (288 – 370) | 5.26 | 0 .154 | 42.9 | -0.04 (-0.09 – 0.02) | 0.2277 |
| Caucasian (4) | 1433  476.08 + 46.13  (434 – 517) | 1980  486.75 + 48.1  (428 – 529) | 5.28 | 0.1525 | 43.2 | -0.05 (-0.12 – 0.01) | 0.1222 |
| AP  Caucasian (2) | 406  425.33 + 148.89 | 406  440 + 149.63 | -- | -- | -- | -- | -- |
|  |  |  |  |  |  |  |  |
| Methionine (2)  mmol/L  Case-Control  CRC  European (2) | 1,980  24.8 + 1.55  (23.7 – 25.9) | 3,513  25.4 + 1.41  (24.4 – 26.4) | 20.0 | <0.0001 | 95 | -0.29 (-0.56 – 0.02) | 0.0303 |
|  |  |  |  |  |  |  |  |
| Vitamin B9 [Folate] (25) nmol/L | 6,212  14.98 + 10.38  (5 – 42.26) | 8,739  14.82 + 9.32  (5 – 41.47) | 444.52 | <0.0001 | 94.6 | -0.08 (-0.23 – 0.08) | 0.3266 |
| Case-Control (14) | 2,248  14.8 + 10.8  (5.02 – 37.8) | 2,958  14.86 + 9.15  (7.22 – 34.6) | 425.92 | <0.0001 | 96.9 | -0.28 (-0.63 – 0.07) | 0.1143 |
| CRC (8) | 1,466  12.92 + 10.99  (5.02 – 37.4) | 1,927  13.04 +9.22  (7.22 – 34.6) | 257.15 | <0.0001 | 97.3 | -0.46 (-0.92 – 0.0) | 0.0511 |
| European (4) | 429  14.89 + 15.07  (5.4 – 37.4) | 873  14.65 +13.3  (8-34.6) | 197.23 | <0.0001 | 98.5 | -1.12 (-2.65 – 0.42) | 0.1547 |
| East Asian (2) | 803  11.0 +8.16  (5.02 – 20.3) | 797  10.54 +4.12  (7.22 – 15.15) | 41.47 | <0.0001 | 95.2 | -0.09 (-0.56 – 0.38) | 0.7093 |
| Middle Eastern (1) | 232  506.3 + 186.6 | 271  461.3 + 217.2 | -- | -- | -- | -- | -- |
| AP (6) | 782  17.32 + 11.1  (7.3 – 37.8) | 1,031  17.3 + 9.29  (8 – 34.6) | 168.49 | <0.0001 | 96.5 | -0.05 (-0.74 – 0.65) | 0.8888 |
| European (2) | 123  22.55 +21.57  (7.3 – 37.8) | 164  21.3 + 18.81  (8 – 34.6) | 145.37 | <0.0001 | 99.3 | 0.05 (-3.44 – 3.55) | 0.9767 |
| Caucasian (2) | 541  11.85 +0.07  (11.8 – 11.9) | 589  13.1 +0.57  (12.7-13.5) | 0.03 | 0.8641 | 0 | -0.17 (-0.29 - -0.06) | 0.0038 |
| East Asian (2) | 118  17.55 + 6.02  (13.29 – 23.9) | 278  17.49 + 3.13 (15.27 – 19.7) | 3.18 | 0.0747 | 68.5 | -0.07 (-0.29 – 0.14) | 0.500 |
| Cohort (11) | 3,964  14.71 + 10.58  (5 – 42.26) | 5,685  14.32 + 10.29  (5 – 41.47) | 15.45 | 0.1164 | 35.3 | -0.03 (-0.07 – 0.01) | 0.1701 |
| CRC (9) | 3,515  12.46 + 5.71  (5 – 21.6) | 5,249  12.06 + 5.34  (5 – 20.7) | 15.23 | 0.0547 | 47.5 | 0.01 (-0.06 – 0.08) | 0.7887 |
| European (3) | 1,645  10.44 + 2.73  (8.86 – 13.6) | 2,603  10.57 + 3.15  (8.56 – 14.2) | 3.24 | 0.198 | 38.2 | -0.08 (-0.14 - -0.01) | 0.0129 |
| Caucasian (5) | 1,582  14.15 + 7.3  (5 – 21.6) | 2,071  13.54 + 6.78  (5 – 20.7) | 6.15 | 0.1884 | 34.9 | 0.0 (-0.06 – 0.07) | 0.8845 |
| East Asian (1) | 288  10 + 15.4 | 575  9.1 + 7.7 | -- | -- | -- | -- | -- |
| AP (2) | 449  24.83 + 24.65  (7.4 – 42.26) | 436  24.53 + 23.95  (7.6 – 41.47) | 0.22 | 0.6408 | 0 | -0.03 (-0.16 – 0.1) | 0.6366 |
|  |  |  |  |  |  |  |  |
| Vitamin B6 (6) nmol/L  Cohort (5) | 3,068  51.51 + 26.46 (24.93 – 96.1) | 5,113  54.78 + 29.53 (24.87 – 105.2) | 3.62 | 0.6043 | 0 | -0.07 (-0.11 – -0.02) | 0.0016 |
| CRC (5) | 2,658  51.5 + 29.57 (24.93 – 96.1) | 4,703  54.88 + 33.03 (24.87 – 105.2) | 2.91 | 0.5722 | 0 | -0.06 (-0.11 – -0.01) | 0.0070 |
| European (3) | 1,643  37.92 + 21.71  (24.93 – 63) | 3,497  38.66 + 24.86  (24.86 – 65) | 1.05 | 0.5887 | 0 | -0.04 (-0.10 – 0.01) | 0.1256 |
| Caucasian (2) | 1,015  71.85 + 34.29  (47.6 – 96.1) | 1,206  79.2 + 36.76  (53.2 – 105.2) | 0.46 | 0.4963 | 0 | -0.10 (-0.19 – 0.02) | 0.0119 |
| AP (1)  Caucasian (1) | 410  49.6 + 34.96 | 410  54.3 + 37.63 | -- | -- | -- | -- | -- |
|  |  |  |  |  |  |  |  |
| Vitamin B2 (4) nmol/L  Cohort | 1,731  12.40 + 5.51  (8.8 – 20.6) | 2,673  12.43 + 5.55  (8.8 – 20.6) | 39.15 | <0.0001 | 92.3 | -0.19 (-0.54 – 0.16) | 0.2898 |
| CRC   European (3) | 1,643  13 + 6.59  (8.8 – 20.6) | 2,597  12.83 + 6.72  (8.8– 20.6) | 3.36 | 0.1858 | 40.6 | 0.00 (-0.05 – 0.07) | 0.7956 |
| AP (1)  European (1) | 88  10.58 + 0.52 | 76  11.23 + 0.76 | -- | -- | -- | -- | -- |

*Notes:* Q = Cochran’s Q; CI = Confidence interval; --: No data
mmol= micromole, pmol= picomole, nmol= nanomole, L=liter
