# Supplementary material for: Meta-analysis of homocysteine-related factors on the risk of colorectal cancer
Source: Oncotarget. 2018 May 22;9(39):25681–97. doi: 10.18632/oncotarget.25355 (PMC5986656; doi:10.18632/oncotarget.25355)
Supplement: Supplementary file 5 [file oncotarget-09-25681-s005.docx]

Supplementary Table 3A: Pooled meta-analysis: Dietary parameters on the risks of colorectal cancer (CRC) and adenomas/polyps (AP) (effect size: 40 studies).

| Dietary factors  (Number of studies) | Case N=14,900  Mean + SD  (Range) | Control  N=149,950  Mean + SD  (Range) | Test of Heterogeneity | | | Test of Association | |
| --- | --- | --- | --- | --- | --- | --- | --- |
|  |  |  | Q | *p* | *I^2^* (%) | Pooled Effect Size  (95% Cl) | *p* |
| Vitamin B12 (17) mcg/day | 6,514  6.45 + 2.78  (2.55 – 11.75) | 8,350  7.02 + 4.85  (2.5 – 23.23) | 185.56 | <0.0001 | 91.40 | -0.06 (-0.17 – 0.05) | 0.3052 |
| Case-Control (12) | 5,344  6.11 + 2.53  (3 – 11.75) | 6,702  6.91 + 5.49  (2.9 – 23.23 | 179.16 | <0.0001 | 93.9 | -0.09 (-0.24 – 0.06) | 0.2647 |
| CRC (8) | 3,337  5.48 + 2.19  (3 – 9.3) | 4,460  5.29 + 2.3  (2.9 – 9.9) | 7.17 | 0.4111 | 2.4 | 0.07 (0.02 – 0.11) | 0.0033 |
| Caucasian (6) | 2,998  5 + 1.82  (3 – 7.5) | 3,965  4.75 + 1.6  (2.9 – 6.7) | 3.35 | 0.6461 | 0 | 0.07 (0.02 – 0.11) | 0.0041 |
| East Asian (1) | 107  9.3 + 3.9 | 224  9.9 + 4.8 | -- | -- | -- | -- | -- |
| Middle Eastern (1) | 232  4.5 + 4.3 | 271  3.9 + 3.5 | -- | -- | -- | -- | -- |
| AP (4) | 2,007  7.29 + 3.02  (4.87 – 11.75) | 2,242  10.15 + 8.77  (4.52 – 23.23) | 167.94 | <0.0001 | 98.2 | -0.49 (-0.97 – 0.0) | 0.0501 |
| European (2) | 825  8.3 + 4.86  (4.87 – 11.74) | 901  13.87 + 13.22  (4.52 – 23.22) | 167.32 | <0.0001 | 99.4 | -1.07 (-3.48 – 1.33) | 0.3822 |
| Caucasian (2) | 1,182  6.47 +0.53  (6.09 – 6.84) | 1,341  6.43 +0.41  (6.14 – 6.72) | 0.19 | 0.66 | 0 | 0.01 (-0.07 – 0.08) | 0.8894 |
| Cohort (5) | 1,170  7.27 + 3.47  (2.55 – 10.7) | 1,648  7.3 + 3.35  (2.5 – 10.6) | 4.12 | 0.39 | 1.9 | -0.02 (-0.1 – 0.05) | 0.5227 |
| CRC (4) | 760  7.51 + 3.96  (2.55 – 10.7) | 1,238  7.53 + 3.82  (2.5 – 10.6) | 4.09 | 0.2514 | 26.7 | -0.02 (-0.11 – 0.07) | 0.6582 |
| European (2) | 278  10.7 + 0 | 278  10.5 + 0.14  (10.4 – 10.6) | 0.04 | 0.8435 | 0 | 0.04 (-0.12 – 0.21) | 0.6026 |
| Caucasian (1) | 194  6.1 + 3.11 | 385  6.6 + 2.81 | -- | -- | -- | -- | -- |
| East Asian (1) | 288  2.55 + 1.5 | 575  2.5 + 1.5 | -- | -- | -- | -- | -- |
| AP (1) | 410  6.3 + 2.81 | 410  6.4 + 3.04 | -- | -- | -- | -- | -- |
|  |  |  |  |  |  |  |  |
| Methionine (17) mg/day | 4,873  1.94 + 0.35  (1.51 – 2.85) | 6,338  1.92 + 0.31  (1.58 – 2.78) | 82.46 | <0.0001 | 80.6 | 0.04 (-0.05 – 0.13) | 0.4098 |
| Case-Control (10) | 3,074  1.99 + 0.44  (1.51 – 2.85) | 3,790  1.95 + 0.39  (1.58 – 2.78) | 52.74 | <0.0001 | 82.9 | 0.1 (-0.03 – 0.22) | 0.12 |
| CRC (5) | 2,181  2.05 + 0.57  (1.51 – 2.85) | 2,575  1.99 + 0.51  (1.58 – 2.78) | 17.90 | 0.0013 | 77.7 | 0.03 (-0.09 – 0.16) | 0.6234 |
| European (1) | 196  2.85 + 1.28 | 200  2.78 + 1.13 | -- | -- | -- | -- | -- |
| Caucasian (4) | 1,985  1.85 + 0.4  (1.51 – 2.4) | 2,375  1.8 + 0.29  (1.58 – 2.2) | 17.66 | 0.0005 | 83 | 0.03 (-0.12 – 0.17) | 0.7194 |
| AP (5) | 893  1.94 + 0.32  (1.6 – 2.45) | 1,215  1.9 + 0.27  (1.62 – 2.32) | 30.47 | <0.0001 | 86.9 | 0.2 (-0.09 – 0.48) | 0.1399 |
| European (1) | 57  2.45 + 0.2 | 192  2.32 + 0.12 | -- | -- | -- | -- | -- |
| Caucasian (4) | 836  1.82 + 0.17  (2 – 7.18) | 1,023  1.79 + 0.15  (1.62 – 1.94) | 0.54 | 0.9111 | 0 | 0.05 (-0.04 – 0.14) | 0.2965 |
| Cohort (7) | 1,799  1.86 + 0.15  (1.7 – 2.12) | 2,548  1.87 + 0.16  (1.6 – 2.05) | 26.85 | 0.0002 | 77.7 | -0.04 (-0.18 – 0.09) | 0.5335 |
| CRC (5) | 833  1.88 + 0.18  (1.7 – 2.12) | 1,581  1.89 + 0.19  (1.6 – 2.05) | 26.29 | <0.0001 | 84.8 | -0.07 (-0.3 – 0.16) | 0.5766 |
| European (2) | 278  1.95 + 0.04  (1.92 – 1.97) | 278  2.0 + 0.06  (1.96 – 2.04) | 2.23 | 0.1355 | 55.1 | -0.12 (-0.29 – 0.05) | 0.1581 |
| Caucasian (1) | 194  1.7 + 0.22 | 385  1.8 + 0.3 | -- | -- | -- | -- | -- |
| East Asian (2) | 361  1.91 + 0.3  (1.7 – 2.12) | 918  1.83 + 0.32  (1.6 – 2.05) | 0.4 | 0.5271 | 0 | 0.15 (0.03 – 0.28) | 0.015 |
| AP (2)  Caucasian (2) | 966  1.8 + 0 | 967  1.8 + 0 | 0 | >0.9999 | -- | -- | -- |
|  |  |  |  |  |  |  |  |
| Vitamin B9 [Folate] (29) mcg/day | 14,900  379.68 + 125.14  (63.7 – 647) | 149,950  373.34 + 83.6  (190 – 638) | 1,393.38 | <0.0001 | 98 | 0.03 (-0.13 – 0.18) | 0.72 |
| Case-Control (19) | 9,454  351.65 + 70.38  (200 – 506.3) | 12,692  361.07 + 66.26  (190 – 471.6) | 104.57 | <0.0001 | 82.8 | -0.05 (-0.12 – 0.03) | 0.2048 |
| CRC (12) | 7,312  367.96 + 72.98  (248 – 506.3) | 10,212  377.59 + 62.1  (263.3 – 471.6) | 46.93 | <0.0001 | 76.6 | -0.07 (-0.14 – 0.0) | 0.0574 |
| European (3) | 2,092  332.88 + 45.2  (288.67 – 379) | 2,884  365.18 + 32.63  (339.87 – 402) | 24.43 | <0.0001 | 91.8 | -0.5 (-1.12 – 0.12) | 0.1136 |
| Caucasian (5) | 3,838  369.78 + 59.22  (320 – 464.9) | 5,406  373.14 + 61.38  (321 – 471.6) | 1.27 | 0.8669 | 0 | -0.04 (-0.08 – 0.0) | 0.0773 |
| East Asian (3) | 1,150  353.9 + 92.28  (248 – 417) | 1,651  369.5 + 93.42  (263.3 – 439) | 0.64 | 0.7256 | 0 | -0.12 (-0.2 - -0.04) | 0.0036 |
| Middle Eastern (1) | 232  506.3 + 186.6 | 271  461.3 + 217.2 | -- | -- | -- | -- | -- |
| AP (7) | 2,142  323.68 + 60.44  (200 – 396) | 2,480  322.56 + 73.61  (200 – 396) | 50.03 | <0.0001 | 88 | 0.0 (-0.2 – 0.2) | 0.9692 |
| European (5) | 960  322.55 + 73.61  (200 – 396) | 1,139  1645.81 + 80.88  (190 – 402) | 30.99 | <0.0001 | 87.1 | 0.01 (-0.33 – 0.37) | 0.9144 |
| Caucasian (2) | 1,182  326.5 + 14.85  (316 – 337) | 1,341  341.75 + 36.41  (316 – 367.5) | 3.68 | 0.055 | 72.8 | -0.08 (-0.23 – 0.07) | 0.3076 |
| Cohort (10) | 5,446  432.94 + 184.4  (63.7 – 647) | 137,258  396.65 + 109.73  (278.33 – 638) | 1,288.48 | <0.0001 | 99.3 | 0.29 (-0.15 – 0.72) | 0.2009 |
| CRC (8) | 4,480  454.46 + 199.8  (63.7 – 647) | 136,291  408.96 + 115.11  (286.67 – 638) | 1,285.49 | <0.0001 | 99.5 | 0.36 (-0.2 – 0.92) | 0.2078 |
| European (2) | 278  647 + 0 | 278  330.5 + 0 | -- | -- | -- | -- | -- |
| Caucasian (4) | 3,790  340.68 + 189.86  (63.7 – 478) | 135,100  407.42 + 88.73  (286.67 – 488) | 609.38 | <0.0001 | 99.5 | -0.86 (-1.48 - -0.24) | 0.0068 |
| East Asian (2) | 412  489.5 + 207.18  (343 – 636) | 913  490.5 + 208.6  (343 – 638) | 0 | 0 .9622 | 0 | 0.0 (-0.12 – 0.11) | 0.974 |
| AP (2)  Caucasian (2) | 966  693.73 + 346.87  (89.85 – 283.33) | 967  347.42 + 97.7  (278.33 – 416.5) | 1.25 | 0.263 | 20.2 | 0.02 (-0.07 – 0.11) | 0.7252 |
|  |  |  |  |  |  |  |  |
| Folate Supplement (3)  mcg/day  Case Control  CRC  Caucasian (3) | 1,637  329.4 + 235.4  (160 – 598.2) | 1,945  360.6 + 192.61  (230 – 581.8) | 1,567.56 | <0.0001 | 99.90 | -2.07 (-4.51 – 0.36) | 0.096 |
|  |  |  |  |  |  |  |  |
| Vitamin B6 (13) mg/day | 5,455  2.14 + 1.1  (1.1 – 5.44) | 7,751  2.32 + 1.7  (1.1 – 7.75) | 178.90 | <0.0001 | 93.30 | -0.14 (-0.28 – 0.00) | 0.063 |
| Case-Control (8) | 4,285  2.21 + 1.41  (1.1 – 5.44) | 6,103  2.48 + 2.18  (1.1 – 7.75) | 167.62 | <0.0001 | 95.8 | -0.22 (-0.44 – -0.01) | 0.0405 |
| CRC (5) | 2,933  1.68 + 0.66  (1.1 – 2.6) | 4,557  1.68 + 0.62  (1.1 – 2.5) | 9.63 | 0.0471 | 58.5 | -0.02 (-0.11 – 0.07) | 0.7061 |
| Caucasian (4) | 2,826  1.73 + 0.75  (1.1 – 2.6) | 4,333  1.7 + 0.71  (1.1 – 2.5) | 1.48 | 0.6877 | 0 | 0.01 (-0.04 – 0.06) | 0.6172 |
| East Asian (1) | 107  1.5 + 0.3 | 224  1.6 + 0.3 | -- | -- | -- | -- | -- |
| AP (3) | 1,352  3.09 + 2.05  (1.65 – 5.44) | 1,546  3.82 + 3.41  (1.59 – 7.75) | 157.97 | <0.0001 | 98.7 | -0.64 (-1.39 – 0.1) | 0.091 |
| European (2) | 825  3.54 + 2.68  (1.65 – 5.44) | 901  4.67 + 4.36  (1.59 – 7.75) | 156.00 | <0.0001 | 99.4 | -1.03 (-3.34 – 1.26) | 0.3762 |
| Caucasian (1) | 526  2.18 + 0.87 | 645  2.13 + 0.9 | -- | -- | -- | -- | -- |
| Cohort (5) | 1,170  2.03 + 0.36  (1.7 – 2.44) | 1,648  2.05 + 0.44  (1.7 – 2.58) | 11.16 | 0.0248 | 64.2 | -0.01 (-0.15 – 0.12) | 0.839 |
| CRC (4) | 760  2.11 + 0.36  (1.8 – 2.44) | 1,238  2.14 + 0.45  (1.7 – 2.58) | 11.12 | 0.0111 | 73 | -0.02 (-0.21 – 0.16) | 0.7909 |
| European (2) | 178  2.42 + 0.03  (2.4 – 2.44) | 178  2.53 + 0.08  (2.47 – 2.58) | 1.89 | 0.1691 | 47.1 | -0.17 (-0.34 – 0.0) | 0.0438 |
| Caucasian (1) | 194  1.8 + 0.3 | 385  1.8 + 0.3 | -- | -- | -- | -- | -- |
| East Asian (1) | 288  1.8 + 0.6 | 575  1.7 + 0.6 | -- | -- | -- | -- | -- |
| AP (1)  Caucasian (1) | 410  1.7 + 0.3 | 410  1.7 + 0.37 | -- | -- | -- | -- | -- |
|  |  |  |  |  |  |  |  |
| Vitamin B2 (7) mg/day | 2,033  3.69 + 4.49  (0.95 – 13.8) | 2,659  3.81 + 4.92  (0.94 – 14.9) | 8.29 | 0.2179 | 27.60 | 0.06 (-0.00 – 0.11) | 0.0536 |
| Case-Control (6) | 1,745  4.15 + 4.74  (1.62 – 13.8) | 2,084  4.29 + 5.21  (1.59 – 14.9) | 8.15 | 0.1481 | 38.7 | 0.06 (0.0 – 0.13) | 0.0561 |
| CRC (4) | 894  5.26 + 5.7  (2.2 – 13.8) | 1,289  5.47 + 6.29  (2 – 14.9) | 7.8 | 0.0503 | 61.5 | 0.04 (-0.11 – 0.2) | 0.6008 |
| European (1) | 36  2.22 + 0.65 | 86  2.28 + 0.94 | -- | -- | -- | -- | -- |
| Caucasian (2) | 751  2.5 + 0.42  (2.2 – 2.8) | 979  2.35 + 0.49  (2 – 2.7) | 1.75 | 0.1858 | 42.90 | 0.12 (0.03 – 0.22) | 0.0094 |
| East Asian (1) | 288  0.95 + 0.3 | 575  0.94 + 0.3 | -- | -- | -- | -- | -- |
| AP (2)  European (2) | 851  1.95 + 0.46  (1.62 – 2.27 | 795  1.94 + 0.49  (1.59 – 2.28) | 0.16 | 0.6919 | 0.00 | 0.05 (-0.05 – 0.14) | 0.3467 |
| Cohort (1)  CRC  Asian (1) | 288  0.95 + 0.3 | 575  0.94 + 0.3 | -- | -- | -- | -- | -- |
|  |  |  |  |  |  |  |  |
| Fiber (14) g/day | 10,309  21.77 + 9.18  (11.17 – 48.1) | 144,548  22.24 + 9.13  (11.5 – 48.4) | 56.56 | <0.0001 | 77.0 | -0.07 (-0.13 – -0.02) | 0.0106 |
| Case-Control (11) | 10,309  24.22 + 8.78  (13.8 – 48.1) | 144,548  24.74 + 8.65  (14.9 – 48.4) | 37.0 | <0.0001 | 73 | -0.06 (-0.13 – 0.0) | 0.049 |
| CRC (7) | 7,691  25.27 + 11.16  (13.8 – 48.1) | 9,695  25.83 + 10.97  (14.9 – 48.4) | 19.0 | 0.0041 | 68.5 | -0.09 (-0.16 - -0.02) | 0.0134 |
| European (1) | 2,028  21.36 + 5.87 | 2,722  22.22 + 2.15 | -- | -- | -- | -- | -- |
| Caucasian (4) | 3,197  23.4 + 4.67 | 4,200  23.83 + 4.66 | 6.68 | 0.0829 | 55.1 | -0.08 (-0.13 – 0.04) | 0.0003 |
|  | (17 – 28.2) | (17.2 – 28.1) |  |  |  |  |  |
| East Asian (1) | 107  13.8 + 6.5 | 224  14.9 + 6.3 | -- | -- | -- | -- | -- |
| Middle Eastern (1) | 232 48.1 + 21.3 | 271  48.4 + 23.3 | -- | -- | -- | -- | -- |
| AP (4) | 2,568  22.39 + 0.96  (21.46 – 23.6) | 4,655  23.84 + 0.92  (21.6 – 23.8) | 7.05 | 0.00702 | 57.5 | -0.01 (-0.07 – 0.05) | 0.6854 |
| European (1) | 768  23.6 + 6.7 | 709  23.1 + 6.7 | -- | -- | -- | -- | -- |
| Caucasian (3) | 1,359  21.99 + 0.64  (21.46 – 22.7) | 1,569  22.75 + 1.1  (21.6 – 23.8) | 2.87 | 0.2384 | 30.3 | -0.06 (-0.13 – 0.02) | 0.1293 |
| Cohort (3)  Caucasian (3) | 2,618  12.79 + 2.78  (11.17 – 16) | 134,853  13.07 + 2.54  (11.5 – 16) | 12.87 | 0.0016 | 84.5 | -0.11 (-0.25 – 0.03) | 0.1264 |
| CRC (2) | 2,177  13.58 + 3.42  (11.17 – 16) | 132,476  13.75 + 3.18  (11.5 – 16) | 10.53 | 0.0012 | 90.5 | -0.13 (-0.4 – 0.15) | 0.3569 |
| AP (1) | 441  11.2 + 4.3 | 2,377  11.7 + 4.8 | -- | -- | -- | -- | -- |
|  |  |  |  |  |  |  |  |
| Vegetables (4) g/day | 2,412  7.15 + 4.57  (2.3 – 12.9) | 4,438  27.61 + 3.96  (2.8 – 12.1) | 438.29 | <0.0001 | 99.3 | -0.29 (-0.95 – 0.36) | 0.3789 |
| Case-Control (3) |  |  |  |  |  |  |  |
| CRC (1)  Caucasian (1) | 548  2.3 + 0.23  (1.3 – 2.7) | 656  2.8 + 0.43  (1.8 – 4.4) | -- | -- | -- | -- | -- |
| AP (2) |  |  |  |  |  |  |  |
| European (1) | 768  12.9 + 5.3 | 709  12.1 + 4.6 | -- | -- | -- | -- | -- |
| Caucasian (1) | 655  8.38 + 7.05 | 696  7.51 + 5.6 | -- | -- | -- | -- | -- |
|  |  |  |  |  |  |  |  |
| Cohort (1)  AP  Caucasian (1) | 411  11.2 + 4.3 | 2,377  11.7 + 4.8 | -- | -- | -- | -- | -- |
|  |  |  |  |  |  |  |  |
| Red Meat (5) g/day | 4,903  59.45 + 10.42  (44.4 – 69) | 137,329  57.18 + 11.74  (41.7 – 67) | 17.96 | 0.0013 | 77.7 | 0.06 (-0.02 – 0.14) | 0.1522 |
| Case-Control (2) | 762  56.44 + 17.02  (44.4 – 68.47) | 920  53.96 + 17.33  (41.7 – 66.21) | 0.25 | 0.6181 | 0 | 0.05 (- 0.05 – 0.15) | 0.334 |
| CRC (1)  East Asian (1) | 107  44.4 + 32.4 | 224  41.7 + 23.5 | -- | -- | -- | -- | -- |
| AP (1)  Caucasian (1) | 655  68.47 + 56.1 | 696  66.21 + 67.4 | -- | -- | -- | -- | -- |
| Cohort (3)  CRC (3) | 4,141  61.45 + 7.55  (53.9 – 69) | 136,409  59.33 + 10.4  (47.5 – 67) | 17.67 | 0.0001 | 88.7 | 0.06 (-0.05 – 0.17) | 0.2933 |
| European (1) | 1,367  53.9 + 39.5 | 2,323  47.5 + 35.5 | -- | -- | -- | -- | -- |
| Caucasian (2) | 2,774  65.25 + 5.3  (61.5 – 69) | 134,086  65.25 + 2.47  (63.5 – 67) | 2.53 | 0.112 | 60.4 | 0.02 (-0.02 – 0.06) | 0.3451 |

*Notes:* Q = Cochran’s Q; CI = Confidence interval; --: No data
