# Supplementary material for: Meta-analysis of homocysteine-related factors on the risk of colorectal cancer
Source: Oncotarget. 2018 May 22;9(39):25681–97. doi: 10.18632/oncotarget.25355 (PMC5986656; doi:10.18632/oncotarget.25355)
Supplement: Supplementary file 6 [file oncotarget-09-25681-s006.docx]

Supplementary Table 3B: Pooled meta-analysis: Dietary parameters on the risks of colorectal cancer (CRC) and adenomas/polyps (AP) (risk ratio: 42 studies)

| Dietary factors  (Number of studies) | Case N=7,979 | Control N=11,561 | Test of Heterogeneity | | | Test of Association | |
| --- | --- | --- | --- | --- | --- | --- | --- |
|  |  |  | Q | *p* | *I^2^* (%) | Risk Ratio (95% Cl) | *p* |
| Vitamin B12 (4), mcg/day  Case-Control  CRC |  |  |  |  |  |  |  |
| Low: <3.5 – 7.3 | 639 | 893 | 0.50 | 0.9189 | 0 | 1.06 (0.98 – 1.15) | 0.128 |
| High: >3.5 – 11.1 | 1,486 | 1,835 | 3.84 | 0.2791 | 21.9 | 1.04 (0.99 – 1.07) | 0.058 |
| Caucasian (3) |  |  |  |  |  |  |  |
| Low: <3.5 – 4.8 | 603 | 821 | 3.93 | 0.1395 | 49.2 | 0.91 (0.83 – 0.99) | 0.0436 |
| High: >3.5 – 7.8 | 1,415 | 1,683 | 3.24 | 0.1974 | 38.4 | 1.04 (1.00 – 1.08) | 0.0424 |
| East Asian (1) |  |  |  |  |  |  |  |
| Low: <7.3 | 36 | 72 | -- | -- | -- | -- | -- |
| High: >7.3 | 71 | 152 | -- | -- | -- | -- | -- |
|  |  |  |  |  |  |  |  |
| Methionine (4), g/day  Case-Control |  |  |  |  |  |  |  |
| Low: <1.4 – 1.89 | 735 | 1,103 | 2.60 | 0.4571 | 0 | 0.98(0.91 – 1.06) | 0.6762 |
| High: >1.4 – 6.5 | 1,468 | 2,138 | 2.11 | 0.549 | 0 | 1.01 (0.96 – 1.04) | 0.7273 |
| CRC (3) |  |  |  |  |  |  |  |
| Caucasian (3) |  |  |  |  |  |  |  |
| Low: <1.4 – 1.89 | 658 | 998 | 2.44 | 0.295 | 18.1 | 0.99 (0.91 – 1.07) | 0.7805 |
| High: >1.4 – 2.5 | 1,370 | 2,015 | 3.31 | 0.1914 | 39.5 | 0.53 (0.49 – 0.56) | <0.0001 |
| AP (1) |  |  |  |  |  |  |  |
| Caucasian (1) |  |  |  |  |  |  |  |
| Low: <1.7 | 77 | 105 | -- | -- | -- | -- | -- |
| High: >1.7 | 98 | 123 | -- | -- | -- | -- | -- |
|  |  |  |  |  |  |  |  |
| Vitamin B9 (Folate), mcg/day |  |  |  |  |  |  |  |
| Low: <169.8 – 484 (14) | 4,631 | 6,660 | 13.93 | 0.3055 | 13.8 | 1.05 (1.02 – 1.07) | 0.0006 |
| High: >282.72 – 508 (13) | 3,348 | 4,931 | 13.92 | 0.3057 | 13.8 | 0.94 (0.91 – 0.98) | 0.0007 |
| Case-Control |  |  |  |  |  |  |  |
| Low: <169.8 – 484 (12) | 3,914 | 6,191 | 13.55 | 0.1943 | 26.2 | 1.05 (1.01 – 1.07) | 0.0013 |
| High: >282.72 – 508 (11) | 2,803 | 4,971 | 13.59 | 0.1924 | 26.4 | 0.94 (0.90 – 0.97) | 0.0015 |
| CRC |  |  |  |  |  |  |  |
| Low: <282.72 – 508 (9) | 3,258 | 4,407 | 22.08 | 0.0048 | 63.8 | 1.07 (0.99 – 1.16) | 0.0812 |
| High: >282.72 – 484 (8) | 2,287 | 3,297 | 19.67 | 0.0063 | 64.4 | 0.95 (0.90 – 1.00) | 0.0728 |
| European |  |  |  |  |  |  |  |
| Low: <169.8 – 305 (3) | 1679 | 2591 | 5.62 | 0.0178 | 82.2 | 1.03 (0.90 – 1.17) | 0.6661 |
| High: >305 – 340 (2) | 1405 | 1893 | 5.88 | 0.0153 | 83 | 0.97 (0.85 – 1.10) | 0.6575 |
| Caucasian (2) |  |  |  |  |  |  |  |
| Low: <253 – 460 | 743 | 892 | 1.15 | 0.2841 | 12.9 | 1.02 (0.96 – 1.07) | 0.5823 |
| High: >388 – 491 | 334 | 420 | 1.11 | 0.291 | 10.3 | 0.97 (0.86 – 1.09) | 0.5826 |
| East Asian (3) |  |  |  |  |  |  |  |
| Low: <169.8 – 484 | 718 | 779 | 0.52 | 0.7705 | 0 | 1.09 (1.03 – 1.16) | 0.0064 |
| High: >282.72 – 484 | 432 | 872 | 2.15 | 0.341 | 7 | 0.89 (0.82 – 0.97) | 0.0072 |
| Middle Eastern (1) |  |  |  |  |  |  |  |
| Low: <320 | 118 | 145 | -- | -- | -- | -- | -- |
| High: >320 | 116 | 112 | -- | -- | -- | -- | -- |
| Cohort (5) |  |  |  |  |  |  |  |
| Low: <242 – 507 | 1,373 | 2,253 | .53 | 0.9707 | 0 | 1.05 (0.99 – 1.10) | 0.0541 |
| High: >375 | 1,061 | 1,634 | .37 | 0.9852 | 0 | 0.94 (0.88 – 0.99) | 0.0491 |
| CRC (3) |  |  |  |  |  |  |  |
| Caucasian |  |  |  |  |  |  |  |
| Low: <242 – 542 | 933 | 1,564 | .18 | 0.9127 | 0 | 1.06 (1.00 – 1.12) | 0.0446 |
| High: >542 | 710 | 1,147 | .01 | 0.9939 | 0 | 0.92 (0.86 – 0.99) | 0.0471 |
| AP (2) |  |  |  |  |  |  |  |
| Caucasian |  |  |  |  |  |  |  |
| Low: <280 – 507 | 440 | 689 | .004 | 0.9497 | 0 | 1.03 (0.94 – 1.12) | 0.5712 |
| High: >508 | 351 | 497 | 0.04 | 0.8386 | 0 | 0.96 (0.86 – 1.08) | 0.5083 |
|  |  |  |  |  |  |  |  |
| Folate Supplement (9)  Case-Control (9) |  |  |  |  |  |  |  |
| No supplement | 2,535 | 3,728 | 31.04 | 0.0001 | 74.2 | 1.04 (0.98 – 1.10) | 0.1468 |
| Supplement | 671 | 873 | 19.77 | 0.0112 | 59.5 | 1.00 (0.82 – 1.23) | 0.9745 |
| CRC (6) |  |  |  |  |  |  |  |
| No supplement | 2,416 | 3,566 | 19.91 | 0.0013 | 74.9 | 1.02 (0.98 – 1.08) | 0.3221 |
| Supplement | 622 | 809 | 14.02 | 0.0155 | 64.3 | 0.99 (0.78 – 1.25) | 0.9232 |
| European (3) |  |  |  |  |  |  |  |
| No supplement | 286 | 315 | 4.81 | 0.0903 | 58.4 | 1.04 (0.99 – 1. 09) | 0.1142 |
| Supplement | 20 | 39 | 3.28 | 0.1938 | 39.1 | 0.66 (0.38 – 1.14) | 0.1374 |
|  |  |  |  |  |  |  |  |
| Caucasian (3) |  |  |  |  |  |  |  |
| No supplement | 2,130 | 3,251 | 12.49 | 0.0019 | 84 | 1.01 (0.94 – 1.09) | 0.6706 |
| Supplement | 602 | 770 | 10.05 | 0.0066 | 80.1 | 1.05 (0.82 – 1.34) | 0.686 |
| AP (3) |  |  |  |  |  |  |  |
| No supplement | 119 | 162 | 10.11 | 0.0064 | 80.2 | 1.02 (0.90 – 1.15) | 0.7514 |
| Supplement | 49 | 64 | 5.96 | 0.0507 | 66.5 | 0.95 (0.69 – 1.30) | 0.7599 |
| European (1) |  |  |  |  |  |  |  |
| No supplement | 260 | 255 | -- | -- | -- | -- | -- |
| Supplement | 18 | 23 | -- | -- | -- | -- | -- |
| Caucasian (2) |  |  |  |  |  |  |  |
| No supplement | 84 | 102 | 1.07 | 0.3008 | 6.6 | 0.93 (0.78 – 1.10) | 0.434 |
| Supplement | 48 | 48 | 0.85 | 0.355 | 0 | 1.13 (0.82 – 1.57) | 0.4306 |
|  |  |  |  |  |  |  |  |
| Vitamin B6 (3), mg/day  Case-Control |  |  |  |  |  |  |  |
| CRC (3) |  |  |  |  |  |  |  |
| Low: <1.46 – 2.55 | 917 | 1,108 | 4.33 | 0.1147 | 53.8 | 1.12 (1.03 – 1.20) | 0.0034 |
| High: >1.46 – 4.08 | 2,158 | 3,029 | 3.27 | 0.1946 | 38.9 | 0.96 (0.92 – 0.98) | 0.0038 |
| European (1) |  |  |  |  |  |  |  |
| Low: <2.55 | 551 | 637 | -- | -- | -- | -- | -- |
| High: >2.55 – 3.26 | 1,477 | 2,085 | -- | -- | -- | -- | -- |
| Caucasian (1) |  |  |  |  |  |  |  |
| Low: <2.44 | 319 | 397 | -- | -- | -- | -- | -- |
| High: >2.44 – 4.08 | 621 | 794 | -- | -- | -- | -- | -- |
| East Asian (1) |  |  |  |  |  |  |  |
| Low: <1.45 | 47 | 74 | -- | -- | -- | -- | -- |
| High: >1.45 – 1.74 | 60 | 150 | -- | -- | -- | -- | -- |
|  |  |  |  |  |  |  |  |
| B6 Supplement (2)  Cohort (2) |  |  |  |  |  |  |  |
| CRC |  |  |  |  |  |  |  |
| European (2) |  |  |  |  |  |  |  |
| No supplement | 247 | 239 | 2.00 | 0.1575 | 49.9 | 1.03 (0.98 – 1.07) | 0.3067 |
| Supplement | 31 | 39 | 2.64 | 0.1043 | 62.1 | 0.79 (0.51 – 1.23) | 0.3077 |
|  |  |  |  |  |  |  |  |
| Vitamin B2 (2), mg/day  Case-Control |  |  |  |  |  |  |  |
| CRC (2) |  |  |  |  |  |  |  |
| Low: <1.49 – 1.84 | 350 | 472 | 2.29 | 0.1301 | 56.4 | 1.01(0.89 – 1.12) | 0.9117 |
| High: >1.49 – 2.68 | 698 | 944 | 2.08 | 0.1488 | 52 | 1.00 (0.94 – 1.05) | 0.9122 |
| Caucasian (1) |  |  |  |  |  |  |  |
| Low: <1.84 | 306 | 398 | -- | -- | -- | -- | -- |
| High: >1.84 – 2.68 | 635 | 794 | -- | -- | -- | -- | -- |
| East Asian (1) |  |  |  |  |  |  |  |
| Low: <1.48 | 44 | 74 | -- | -- | -- | -- | -- |
| High: >1.49 – 1.85 | 63 | 150 | -- | -- | -- | -- | -- |
|  |  |  |  |  |  |  |  |
| Red Meat (5) |  |  |  |  |  |  |  |
| Low: <1 serving/day | 917 | 1,432 | 5.87 | 0.2092 | 31.8 | 0.97 (0.93 – 1.01) | 0.1711 |
| High: >1 serving/day | 340 | 471 | 4.95 | 0.2927 | 19.2 | 1.09 (0.96 – 1.23) | 0.1714 |
| Case Control (1)  South Asian (1) |  |  |  |  |  |  |  |
| Low: <0.8 times/day | 205 | 429 | -- | -- | -- | -- | -- |
| High: >0.8 times/day | 143 | 124 | -- | -- | -- | -- | -- |
| Cohort (4)  Caucasian (4) |  |  |  |  |  |  |  |
| Low: <1 serving/day | 834 | 1,335 | 3.15 | 0.3691 | 4.7 | 0.98 (0.94 – 1.03) | 0.4144 |
| High: >1 serving/day | 293 | 438 | 2.8 | 0.4229 | 0 | 1.06 (0.93 – 1.2) | 0.4147 |
| Low: <1 serving/day | 423 | 906 | 1.81 | 0.4032 | 0 | 1.00 (0.94 – 1.06) | 0.9739 |
| High: >1 serving/day | 150 | 314 | 1.85 | 0.3946 | 0 | 0.99 (0.84 – 1.17) | 0.974 |
| AP (1) |  |  |  |  |  |  |  |
| Low: <1 serving/day | 411 | 429 | -- | -- | -- | -- | -- |
| High: >1 serving/day | 143 | 124 | -- | -- | -- | -- | -- |
|  |  |  |  |  |  |  |  |
| Multivitamins (19) |  |  |  |  |  |  |  |
| No supplement | 4,600 | 5,731 | 81.94 | <0.0001 | 78 | 1.04 (0.99 – 1.09) | 0.0845 |
| Supplement | 2,494 | 3,674 | 84.48 | <0.0001 | 78.7 | 0.94 (0.87 – 1.01) | 0.108 |
| Case-Control (9) |  |  |  |  |  |  |  |
| No supplement | 2,531 | 2,755 | 40.04 | <0.0001 | 80 | 1.0 (0.95 – 1.06) | 0.9155 |
| Supplement | 1,246 | 1,541 | 38.77 | <0.0001 | 79.4 | 0.99 (0.89 – 1.09) | 0.7974 |
| CRC (6) |  |  |  |  |  |  |  |
| No supplement | 1,504 | 1,779 | 34.23 | <0.0001 | 85.4 | 0.98 (0.91 – 1.06) | 0.6432 |
| Supplement | 609 | 832 | 28.79 | <0.0001 | 82.6 | 1.02 (0.88 0 1.19) | 0.7601 |
| Caucasian (3) |  |  |  |  |  |  |  |
| No supplement | 543 | 612 | 26.37 | <0.0001 | 92.4 | 0.96 (0.8 – 1.15) | 0.6696 |
| Supplement | 408 | 593 | 24.42 | <0.0001 | 91.8 | 1.04 (0.86 – 1.26) | 0.7049 |
| East Asian (3) |  |  |  |  |  |  |  |
| No supplement | 961 | 1,167 | 2.91 | 0.2334 | 31.3 | 1.0 (0.97 – 1.05) | 0.688 |
| Supplement | 201 | 239 | 3.06 | 0.2164 | 34.7 | 0.96 (0.81 – 1.15) | 0.6848 |
| AP (3) |  |  |  |  |  |  |  |
| No supplement | 1,027 | 976 | 5.71 | 0.0574 | 65 | 1.04 (0.99 – 1.09) | 0.1609 |
| Supplement | 637 | 709 | 3.48 | 0.1756 | 42.5 | 0.95 (0.88 0 1.02) | 0.1622 |
| European (1) |  |  |  |  |  |  |  |
| No supplement | 634 | 582 | -- | -- |  | -- | -- |
| Supplement | 134 | 127 | -- | -- |  | -- | -- |
| Caucasian (2) |  |  |  |  |  |  |  |
| No supplement | 393 | 394 | 3.31 | 0.0688 | 69.8 | 1.09 (0.98 – 1.21) | 0.1234 |
| Supplement | 503 | 582 | 3.41 | 0.0649 | 70.7 | 0.94 (0.87 – 1.02) | 0.1252 |
| Cohort (9) |  |  |  |  |  |  |  |
| No supplement | 1,759 | 2,683 | 48.68 | <0.0001 | 83.6 | 1.1 (1.0 – 1.2) | 0.0333 |
| Supplement | 1,004 | 1,873 | 31.79 | 0.0001 | 74.8 | 0.89 (0.78 – 1.01) | 0.0706 |
| CRC (7) |  |  |  |  |  |  |  |
| No supplement | 1,195 | 2,144 | 51.23 | <0.0001 | 88.3 | 1.13 (1.0 – 1.27) | 0.0423 |
| Supplement | 596 | 1,449 | 29.23 | <0.0001 | 79.5 | 0.86 (0.72 – 1.03) | 0.1094 |
| Caucasian (6) |  |  |  |  |  |  |  |
| No supplement | 938 | 1,606 | 25.59 | 0.0001 | 80.5 | 1.17 (1.03 – 1.32) | 0.0135 |
| Supplement | 565 | 1,412 | 21.14 | 0.0008 | 76.4 | 0.81 (0.69 – 0.96) | 0.0153 |
| East Asian (1) |  |  |  |  |  |  |  |
| No supplement | 288 | 575 | -- | -- |  | -- | -- |
| Supplement | 554 | 553 | -- | -- |  | -- | -- |
| AP (2)  Caucasian (2) |  |  |  |  |  |  |  |
| No supplement | 564 | 539 | 0.08 | 0.7726 | 0 | 1.05 (0.97 – 1.13) | 0.2593 |
| Supplement | 408 | 424 | 0.37 | 0.5446 | 0 | 0.96 (0.87 – 1.06) | 0.4486 |

*Notes:* Q = Cochran’s Q; CI = Confidence interval; --: No data
