# Supplementary material for: Meta-analysis of homocysteine-related factors on the risk of colorectal cancer
Source: Oncotarget. 2018 May 22;9(39):25681–97. doi: 10.18632/oncotarget.25355 (PMC5986656; doi:10.18632/oncotarget.25355)
Supplement: Supplementary file 7 [file oncotarget-09-25681-s007.docx]

Supplementary Table 4A: Pooled meta-analysis: Lifestyle factors on the risks of colorectal cancer and adenomas/polyps (effect size: 25 studies).

| Lifestyle factors  (Number of studies) | Case N=13,086  Mean + SD  (Range) | Control  N=146,909  Mean + SD  (Range) | Test of Heterogeneity | | | Test of Association | |
| --- | --- | --- | --- | --- | --- | --- | --- |
|  |  |  | Q | *p* | *I^2^* (%) | Pooled Effect Size  (95% Cl) | *p* |
| Alcohol (23) g/day | 13,086  9.61 + 6.58  (0.3 – 25.17) | 146,909  6.75 + 5.38  (0.3 – 19.14) | 132.71 | <0.0001 | 83.4 | 0.1 (0.04 – 0.16) | 0.0013 |
| Case-Control (15) | 8,676  10.59 + 7.56  (0.3 – 25.17) | 10,818  6.34 + 6.06  (0.3 – 19.14) | 121.43 | <0.0001 | 88.5 | 0.17 (0.08 – 0.27) | 0.0004 |
| CRC (9) | 6,433  10.69 + 8.25  (0.3 – 25.17) | 8,336  8.31 + 6.9  (0.3 – 19.14) | 32.62 | <0.0001 | 75.5 | 0.05 (-0.03 – 0.13) | 0.1864 |
| European (3) | 2,252  13.68 + 11.16  (2.89 – 25.17) | 2,998  8.03 + 9.63  (2 – 19.14) | 28.32 | <0.0001 | 92.9 | 0.36 (-0.07 – 0.78) | 0.0995 |
| Caucasian (5) | 4,123  7.08 + 5.49  (0.3 – 14.7) | 5,187  6.6 + 4.8  (0.3 – 12) | 4.02 | 0.4039 | 0.4 | 0.02 (-0.02 – 0.6) | 0.3684 |
| East Asian (1) | 58  19.8 + 29.6 | 151  17.7 + 28.2 | -- | -- | -- | -- | -- |
| AP (6) | 2,243  10.45 + 7.16  (3.3 – 24) | 2,482  3.4 + 3.07  (0.57 – 7.93) | 50.16 | <0.0001 | 90 | 0.37 (0.16 – 0.58) | 0.0005 |
| European (3) | 820  13.16 + 9.54  (6 – 24) | 861  1.52 + 0.82  (0.57 – 2) | 32.76 | <0.0001 | 93.9 | 0.77 (-0.06 – 1.61) | 0.0709 |
| Caucasian (3) | 1,423  7.73 + 3.84  (3.3 – 10.1) | 1,621  5.28 + 3.51  (1.3 – 7.93) | 4.47 | 0.107 | 55.3 | 0.15 (0.08 – 0.22) | <0.0001 |
| East Asian (1) | 58  19.8 + 29.6 | 151  17.7 + 28.2 | -- | -- | -- | -- | -- |
| Cohort (8) | 4,410  7.78 + 3.95  (0.8 – 13.1) | 136,091  7.51 + 4.06  (0.4 – 13.9) | 5.59 | 0.5888 | 0 | 0.01 (-0.02 – 0.05) | 0.482 |
| CRC (6) | 3,444  8.12 + 4.61  (0.8 – 13.1) | 135,124  7.68 + 4.79  (0.4 – 13.9) | 3.88 | 0.5665 | 0 | 0.02 (-0.02 – 0.06) | 0.3131 |
| European (2) | 278  11.95 + 1.63  (10.8 – 13.1) | 278  12.4 + 2.12  (10.9 + 13.9) | 0.06 | 0.8014 | 0 | -0.03 (0.19 – 0.14) | 0.7644 |
| Caucasian (4) | 3,166  6.2 + 4.46  (0.8 – 11.4) | 134,846  5.33 + 3.81  (0.4 – 9.6) | 3.53 | 0.3163 | 15.1 | 0.02 (-0.02 – 0.06) | 0.2709 |
| AP (2)  Caucasian (2) | 966  6.75 + 0.35  (6.5 – 7) | 967  7 + 0.28  (6.8 – 7.2) | 0.81 | 0.3691 | 0 | -0.03 (-0.12 – 0.06) | 0.541 |
|  |  |  |  |  |  |  |  |
| Smoking, Caucasian (9)  pack-year | 4,840  14.94 + 6.78  (3.9 – 27) | 6,895  11.32 + 6.97  (1 – 22) | 9.79 | 0.2801 | 18.30 | 0.16 (0.11 – 0.19) | <0.0001 |
| Case-Control (3) | 1,444  17.94 + 9.53  (8 – 27) | 1,632  13.28 + 10.94  (1 – 22) | 2.24 | 0.3264 | 10.7 | 0.12 (0.05 – 0.19) | 0.0006 |
| CRC (1) | 548  8 + 50.41 | 656  1 + 34.1 | -- | -- | -- | -- | -- |
| AP (2) | 896  22.92 + 5.77  (18.83 – 27) | 976  19.43 + 3.64  (16.85 – 22) | 1.39 | 0.2387 | 28 | 0.1 (0.01 – 0.19) | 0.0364 |
| Cohort (6) | 3,396  13.42 + 5.39  (3.9 – 19.1) | 4,963  10.33 + 5.13  (2 – 16.2) | 6.45 | 0.2646 | 22.5 | 0.17 (0.12 – 0.19) | <0.0001 |
| CRC (5) | 2,986  12.52 + 5.48  (3.9 – 19.1) | 4,553  9.36 + 5.08  (2 – 16.2 | 6.08 | 0.1931 | 34.2 | 0.17 (0.05 – 0.19) | <0.0001 |
| AP (1) | 410  18 + 22 | 410  15.2 + 21.7 | -- | -- | -- | -- | -- |

*Notes:* Q = Cochran’s Q; CI = Confidence interval; --: No data
