# Supplementary material for: Meta-analysis of homocysteine-related factors on the risk of colorectal cancer
Source: Oncotarget. 2018 May 22;9(39):25681–97. doi: 10.18632/oncotarget.25355 (PMC5986656; doi:10.18632/oncotarget.25355)
Supplement: Supplementary file 8 [file oncotarget-09-25681-s008.docx]

Supplementary Table 4B: Pooled meta-analysis: Lifestyle on the risks of colorectal cancer and adenomas/polyps (risk ratio: 61 studies).

| Lifestyle factors  (Number of studies) | Case N=26,107 | Control N=163,231 | Test of Heterogeneity | | | Test of Association | |
| --- | --- | --- | --- | --- | --- | --- | --- |
|  |  |  | Q | *p* | *I^2^* (%) | Risk Ratio (95% Cl) | *p* |
| Alcohol |  |  |  |  |  |  |  |
| Non-drinker (25) | 4,806 | 8,006 | 131.97 | <0.0001 | 81.8 | 0.97 (0.91 – 1.03) | 0.2676 |
| Drinker or 1 – 30 g/day (32) | 10,239 | 15,337 | 126.19 | <0.0001 | 81 | 1.01 (0.97 – 1.06) | 0.4339 |
| Case-Control |  |  |  |  |  |  |  |
| Non-drinker (19) | 3,593 | 4,340 | 124.97 | <0.0001 | 85.6 | 0.95 (0.86 – 1.04) | 0.2485 |
| Drinker (22) | 6,210 | 8,387 | 122.04 | <0.0001 | 85.3 | 1.02 (0.95 – 1.09) | 0.5984 |
| CRC |  |  |  |  |  |  |  |
| Non-drinker (13) | 2,852 | 3,402 | 87.71 | <0.0001 | 86.3 | 1.01 (0.91 – 1.12) | 0.9231 |
| Drinker or 1 – 30 g/day (15) | 4,284 | 6,290 | 108.17 | <0.0001 | 88.9 | 0.97 (0.88 – 1.07) | 0.5743 |
| European |  |  |  |  |  |  |  |
| Non-drinker (4) | 230 | 425 | 7.73 | 0.052 | 61.2 | 0.82 (0.71 – 0.94) | 0.0033 |
| Drinker or 1 – 30 g/day (5) | 810 | 1,650 | 12.03 | 0.0073 | 75.1 | 1.06 (0.92 – 1.22) | 0.3962 |
| Caucasian (2) |  |  |  |  |  |  |  |
| Non-drinker | 543 | 961 | 0.49 | 0.4847 | 0 | 0.93 (0.86 – 1.02) | 0.1271 |
| Drinker or 1 – 20 g/day or   >8 drinks/week (8) | 1,249 | 2,002 | 0.48 | 0.4896 | 0 | 1.03 (0.99 – 1.07) | 0.1225 |
| East Asian |  |  |  |  |  |  |  |
| Non-drinker (5) | 1,760 | 1,694 | 22.51 | 0.0002 | 82.2 | 1.12 (0.99 – 1.26) | 0.0707 |
| Drinker or 1 – 30 g/day (6) | 2,112 | 2,539 | 52.02 | <0.0001 | 92.3 | 0.83 (0.67 – 1.03) | 0.0844 |
| Middle Eastern (1) |  |  |  |  |  |  |  |
| Non-drinker | 118 | 145 | -- | -- | -- | -- | -- |
| Drinker or >325 mcg/day | 116 | 112 | -- | -- | -- | -- | -- |
| AP |  |  |  |  |  |  |  |
| Non-drinker (6) | 741 | 938 | 9.24 | 0.0997 | 45.9 | 0.86 (0.79 – 0.93) | 0.0001 |
| Drinker or 1 – 80 g/day (7) | 1,926 | 2,097 | 9.12 | 0.1043 | 45.2 | 1.07 (1.02 – 1.12) | 0.0021 |
| European (2) |  |  |  |  |  |  |  |
| Non-drinker | 71 | 104 | 2.32 | 0.1278 | 56.9 | 0.73 (0.56 – 0.96) | 0.026 |
| Drinker or 1 – 20 g/day | 273 | 266 | 6.03 | 0.014 | 83.4 | 1.17 (0.84 – 1.64) | 0.3457 |
| Caucasian (3) |  |  |  |  |  |  |  |
| Non-drinker | 474 | 639 | 5.09 | 0.0784 | 60.7 | 0.86 (0.78 – 0.94) | 0.0008 |
| Drinker or 1 – 7 g/day,  >3 drinks/week | 704 | 739 | 0.64 | 0.7256 | 0 | 1.12 (1.03 – 1.18) | 0.0046 |
| East Asian |  |  |  |  |  |  |  |
| Non-drinker (1) | 196 | 195 | -- | -- | -- | -- | -- |
| Drinker or 1 – 80 g/day (2) | 949 | 1,092 | -- | -- | -- | -- | -- |
| Cohort |  |  |  |  |  |  |  |
| Non-drinker (7) | 1,416 | 3,880 | 2.23 | 0.8969 | 0 | 0.97 (0.93 – 1.02) | 0.2271 |
| Drinker (11) | 3,736 | 6,529 | 11.44 | 0.1204 | 38.8 | 1.02 (0.99 – 1.04) | 0.0646 |
| CRC |  |  |  |  |  |  |  |
| Non-drinker (5) | 813 | 1,444 | 1.73 | 0.7848 | 0 | 0.98 (0.92 – 1.05) | 0.6291 |
| Drinker (9) | 3,344 | 6,034 | 5.18 | 0.3948 | 3.4 | 1.01 (0.99 – 1.04) | 0.2148 |
| European |  |  |  |  |  |  |  |
| Non-drinker (1) | 323 | 1,166 | -- | -- | -- | -- | -- |
| Drinker (2) | 2,924 | 2,354 | 6.02 | 0.0142 | 83.4 | 1.07 (0.87 – 1.32) | 0.5207 |
| Caucasian |  |  |  |  |  |  |  |
| Non-drinker (3) | 1,172 | 1,045 | 0.41 | 0.8163 | 0 | 0.97 (0.90 – 1.06) | 0.5289 |
| Drinker, >8 drinks/week (6) | 3,088 | 3,572 | 0.16 | 0.9248 | 0 | 1.01 (0.97 – 1.05) | 0.5259 |
| East Asian (1) |  |  |  |  |  |  |  |
| Non-drinker | 1,851 | 223 | -- | -- | -- | -- | -- |
| Drinker | 2,147 | 109 | -- | -- | -- | -- | -- |
| AP (2) |  |  |  |  |  |  |  |
| Caucasian |  |  |  |  |  |  |  |
| Non-drinker | 603 | 2,436 | 0.18 | 0.6691 | 0 | 0.96 (0.92 – 1.01) | 0.1017 |
| Drinker, >3 drinks/week | 392 | 494 | 3.68 | 0.0552 | 72.8 | 1.08 (0.99 – 1.19) | 0.089 |
|  |  |  |  |  |  |  |  |
| Smoking (51) |  |  |  |  |  |  |  |
| Never/Former | 18,075 | 89,088 | 236.34 | <0.0001 | 78.7 | 0.97 (0.95 – 0.99) | 0.0105 |
| Current | 8,032 | 74,143 | 255.08 | <0.0001 | 77.8 | 1.09 (1.03 – 1.15) | 0.0031 |
| Case-Control (42)  CRC (31) |  |  |  |  |  |  |  |
| Never/Former | 13,141 | 81,289 | 89.0 | <0.0001 | 66.3 | 1.01 (0.99 – 1.03) | 0.4551 |
| Current | 5,567 | 71,567 | 84.93 | <0.0001 | 64.7 | 0.99 (0.94 – 1.05) | 0.9456 |
| European (10) |  |  |  |  |  |  |  |
| Never/Former | 6,217 | 7,747 | 37.92 | <0.0001 | 76.3 | 1.03 (0.99 – 1.06) | 0.1423 |
| Current | 1,898 | 2,397 | 29.76 | 0.0005 | 69.8 | 0.95 (0.84 – 1.07) | 0.3636 |
| Caucasian (6) |  |  |  |  |  |  |  |
| Never/Former | 3,527 | 69,877 | 15.08 | 0.01 | 66.8 | 0.99 (0.96 – 1.04) | 0.9807 |
| Current | 1,679 | 67,087 | 14.06 | 0.0153 | 64.4 | 0.99 (0.9 – 1.12) | 0.9405 |
|  |  |  |  |  |  |  |  |
| Hispanic (1) |  |  |  |  |  |  |  |
| Never/Former | 372 | 358 | -- | -- | -- | -- | -- |
| Current | 444 | 457 | -- | -- | -- | -- | -- |
| East Asian (9) |  |  |  |  |  |  |  |
| Never/Former | 2,585 | 2,950 | 23.97 | 0.0023 | 66.6 | 1.0 (0.94 – 1.06) | 0.9486 |
| Current | 1,544 | 1,746 | 22.61 | 0.0039 | 64.6 | 1.01 (0.91 – 1.13) | 0.8637 |
| South Asian (3) |  |  |  |  |  |  |  |
| Never/Former | 365 | 396 | 3.94 | 0.139 | 49.3 | 0.97 (0.90 – 1.05) | 0.5339 |
| Current | 153 | 185 | 2.31 | 0.3144 | 13.6 | 1.05 (0.89 – 1.23) | 0.5258 |
| Middle Eastern (2) |  |  |  |  |  |  |  |
| Never/Former | 259 | 216 | 4.97 | 0.0258 | 79.9 | 0.96 (0.75 – 1.23) | 0.7501 |
| Current | 112 | 85 | 4.49 | 0.034 | 77.8 | 1.04 (0.61 – 1.77) | 0.8841 |
| AP (11) |  |  |  |  |  |  |  |
| Never/Former | 2,314 | 3,171 | 43.13 | <0.0001 | 76.8 | 0.86 (0.8 – 0.91) | <0.0001 |
| Current | 1,682 | 1,405 | 44.99 | <0.0001 | 77.8 | 1.44 (1.25 – 1.65) | <0.0001 |
| European (4) |  |  |  |  |  |  |  |
| Never/Former | 839 | 1,033 | 3.16 | 0.2056 | 36.8 | 0.91 (0.88 – 0.94) | <.0001 |
| Current | 198 | 138 | 0.85 | 0.6526 | 0 | 1.64 (1.34 – 2.01) | <.0001 |
| Caucasian (3) |  |  |  |  |  |  |  |
| Never/Former | 520 | 918 | 4.89 | 0.1797 | 38.7 | 0.79 (0.73 – 0.86) | <0.0001 |
| Current | 743 | 743 | 5.48 | 0.1401 | 42.5 | 1.23 (1.15 – 1.32) | <0.0001 |
| East Asian (4) |  |  |  |  |  |  |  |
| Never/Former | 520 | 918 | 7.80 | 0.099 | 48.8 | 0.79 (0.72 – 0.85) | <0.0001 |
| Current | 743 | 743 | 7.47 | 0.1127 | 46.5 | 1.23 (1.15 – 1.31) | <0.0001 |
| Cohort (8) |  |  |  |  |  |  |  |
| Never/Former | 2,620 | 4,628 | 16.11 | 0.0241 | 56.6 | 0.97 (0.94 – 1.01) | 0.1455 |
| Current | 783 | 1,171 | 8.41 | 0.2982 | 16.7 | 1.14 (1.05 – 1.23) | 0.0013 |
| CRC (7) |  |  |  |  |  |  |  |
| Never/Former | 2,217 | 4,197 | 12.84 | 0.0456 | 53.3 | 0.98 (0.95 – 1.02) | 0.3308 |
| Current | 637 | 1,059 | 7.09 | 0.3129 | 15.3 | 1.11 (1.02 – 1.21) | 0.0119 |
| European (1) |  |  |  |  |  |  |  |
| Never/Former | 1,012 | 1,801 | -- | -- | -- | -- | -- |
| Current | 346 | 511 | -- | -- | -- | -- | -- |
| Caucasian (4) |  |  |  |  |  |  |  |
| Never/Former | 958 | 1817 | 1.35 | 0.7177 | 0 | 0.99 (0.96 – 1.02) | 0.4202 |
| Current | 124 | 211 | 1.13 | 0.7695 | 0 | 1.09 (0.89 – 1.34) | 0.4134 |
| East Asian (2) |  |  |  |  |  |  |  |
| Never/Former | 247 | 579 | 8.64 | 0.0033 | 88.4 | 0.99 (0.77 – 1.28) | 0.9301 |
| Current | 167 | 337 | 5.80 | 0.016 | 82.8 | 0.95 (0.58 – 1.55) | 0.8325 |
| AP (1) |  |  |  |  |  |  |  |
| Caucasian (1) |  |  |  |  |  |  |  |
| Never/Former | 403 | 431 | -- | -- | -- | -- | -- |
| Current | 146 | 112 | -- | -- | -- | -- | -- |

*Notes:* Q = Cochran’s Q; CI = Confidence interval; --: No data
